# Learning physical characteristics like animals for legged robots

**DOI:** 10.1093/nsr/nwad045

**Published:** 2023-02-22

**Authors:** Peng Xu, Liang Ding, Zhengyang Li, Huaiguang Yang, Zhikai Wang, Haibo Gao, Ruyi Zhou, Yang Su, Zongquan Deng, Yanlong Huang

**Affiliations:** Key Laboratory of Robotics and Systems, Harbin Institute of Technology, Harbin 150001, China; Key Laboratory of Robotics and Systems, Harbin Institute of Technology, Harbin 150001, China; Key Laboratory of Robotics and Systems, Harbin Institute of Technology, Harbin 150001, China; Key Laboratory of Robotics and Systems, Harbin Institute of Technology, Harbin 150001, China; Key Laboratory of Robotics and Systems, Harbin Institute of Technology, Harbin 150001, China; Key Laboratory of Robotics and Systems, Harbin Institute of Technology, Harbin 150001, China; Key Laboratory of Robotics and Systems, Harbin Institute of Technology, Harbin 150001, China; Key Laboratory of Robotics and Systems, Harbin Institute of Technology, Harbin 150001, China; Key Laboratory of Robotics and Systems, Harbin Institute of Technology, Harbin 150001, China; School of Computing, University of Leeds, Leeds LS29JT, UK

**Keywords:** legged robot, unsupervised learning, environmental perception, cognitive learning

## Abstract

Physical characteristics of terrains, such as softness and friction, provide essential information for legged robots to avoid non-geometric obstacles, like mires and slippery stones, in the wild. The perception of such characteristics often relies on tactile perception and vision prediction. Although tactile perception is more accurate, it is limited to close-range use; by contrast, establishing a supervised or self-supervised contactless prediction system using computer vision requires adequate labeled data and lacks the ability to adapt to the dynamic environment. In this paper, we simulate the behavior of animals and propose an unsupervised learning framework for legged robots to learn the physical characteristics of terrains, which is the first report to manage it online, incrementally and with the ability to solve cognitive conflicts. The proposed scheme allows robots to interact with the environment and adjust their cognition in real time, therefore endowing robots with the adaptation ability. Indoor and outdoor experiments on a hexapod robot are carried out to show that the robot can extract tactile and visual features of terrains to create cognitive networks independently; an associative layer between visual and tactile features is created during the robot’s exploration; with the layer, the robot can autonomously generate a physical segmentation model of terrains and solve cognitive conflicts in an ever-changing environment, facilitating its safe navigation.

## Teaser

Legged robots learn to perceive physical characteristics of terrains online and incrementally, while resolving cognitive conflicts.

## INTRODUCTION

With the development of deep learning, robot mobility, and simultaneous localization and mapping techniques, mobile robots are able to move from laboratories to outdoor environments [1–3]. Such progress is particularly evident in legged robots, whose maneuverability with discrete footholds allows them to operate in the wild, planetary or deep-sea environments [4–6]. One of the most important challenges associated with outdoor tasks is that robots need to tackle ever-changing environments, which is not only reflected in various geometric shapes but also in diverse physical parameters of terrains. Until now, robot navigation has largely relied on geometric characteristics of terrains, such as roughness, height and slope [7,8]. However, geometric features may not suffice for safe navigation in the wild. For example, the robot could be stuck in soft terrain or lose balance in slippery terrain. In order to avoid irreversible failures [9], an appealing solution is to exploit physical characteristics of terrains, such as softness and friction. In fact, it is challenging to study physical characteristics in diverse and dynamic environments. As a result of changes in weather, light, humidity and terrain material, terrains with similar appearances may differ significantly in their physical characteristics; or terrains with disparate appearances could share similar physical properties. Such changes in the environment may also lead to cognition conflicts, e.g. what appears to be a passable weedy surface turns out to be very muddy below because of rain, which further deteriorates the perception of the robot. Although it is difficult to realize the autonomous navigation of robots in a complex environment, adapting to dynamic hazards seems to be effortless for animals [10].

Animals are able to establish their fear mechanism to evade dangers through observing and interacting with the environment [10,11]. For example, after hitting glass a number of times, an orangutan can learn to keep its distance from the glass; when a cat walks in a dangerous area, it uses its feet to test the bearing capacity of the terrain and avoids soft regional topography. In order to build their cognition systems, animals first need to interact with the environment in a proactive fashion so that they can explore the environment sufficiently and gain more useful experiences. Then the tactile and visual perception ability helps them to collect environmental data. Tactile perception ability is essential for animals to perceive dangers accurately, which transmits various sensory information such as pain, temperature, pressure and vibration to their nervous system, helping them perceive surroundings and avoid potential harm [12]. On the other hand, vision contains rich information, such as texture, color and illumination, helping organisms remotely extract the characteristics of the environment and predict dangers. Besides, when several modalities appear at the same time, brains will establish internal connections among the modalities, endowing animals with the ability to establish connections among different perceptions. For example, humans who have been bitten by snakes may imagine a sense of pain even when they see an object with a snake shape [13]. Apart from collecting perceptual information and interactive experiences, updating their cognitive system dynamically by constantly summarizing experiences, updating cognition and solving conflicts is another essential ability for them to adapt to dynamic changes in the environment, which is also a key characteristic of highly intelligent animals [14]. In this way, animals can adapt to the dynamic environment by creating and updating their cognition systems actively. Thus, it is desirable for robots to act like animals: learning physical characteristics of the environment.

Before imitating the behavior of animals, the first question that comes to mind is what environmental features the legged robot needs to extract to avoid environmental dangers. The geometric features of the environment are the first consideration of robots, as most obstacles, like slopes, stumbling blocks and steep terrains, can be detected by these features. For the legged robot, geometric features such as roughness, slope and the step degree of elevation maps are the main factors considered in path planning and foothold selection [8,15]. However, it is not practical to distinguish a flat swamp with a totally different softness degree from a hard flagstone floor just by its geometric appearance, but ignoring the difference may lead to irreparable loss [9]. Extracting semantic clues from images, such as designing many features like color and frequency characteristics, to distinguish terrains seems to be a feasible solution [16,17]. Notably, constructing semantic segmentation networks through machine learning methods has shown better efficiency and advantages in semantic feature extraction recently [18,19]. On another front, energy consumption is another concern in measuring the risk of terrains. In order to plan a path consuming minimal energy for robots, predicting the cost of the transport map of the environment through remote sensors like cameras is investigated to promote the robot’s navigation [20,21]. However, in the field environment, the physical characteristics of terrains greatly influence robot traversability. Although semantic clues through vision can help robots avoid non-geometric obstacles, they cannot reveal more about the specific parameters of a terrain, which limits their application; e.g. a semantic picture cannot assist the robot to determine whether the friction coefficient of a terrain can meet its demand of 1 m/s velocity planning.

Since physical features of terrains are crucially important, how should a robot extract them? Two of the most concerning terrain physical parameters for mobile robots are softness and friction [22]. Softness is required when estimating whether a terrain has sufficient carrying capacity, while friction is crucial for driving force. Tactile perception is the most direct way to extract physical parameters through contacting terrains. For instance, in the planetary exploration field, a planetary vehicle first collects contact data during wheel rotation and then it can identify terrain parameters like soil shear parameters based on terramechanic models [23,24]. For legged robots, physical parameters of terrains can be extracted through normal contact and tangential interaction between feet and terrains [22,25,26]. In addition, coarse-grained estimation of physical parameters by classifying them into categories is a more common practice in the robotics field. There are many tactile perception works based on different sensor information, such as force and moment [27], vibration [28], execution errors [29], etc. It is worth mentioning that the fusion of multiple sensors can achieve higher accuracy, and much work has shown its advantages [30,31]. Although the haptic approach provides the most reliable data on the physical features of the terrain, it becomes powerless when performing remote planning. A natural biological process emerges: feel, observe and predict.

Imagine you walk to a frozen lake, you raise a foot and stomp on the ice a few times, and then whether the area with a similar appearance as the touch point is safe can be determined. This seems to be an ideal solution to predict physical characteristics through vision. Several works have tried to realize it by using computer vision technologies to predict the physical parameters of terrains by establishing a mapping relationship between tactile characteristics and semantic classes using supervised methods [32–35]. Nevertheless, they require a large number of human annotations and hence become less applicable in ever-changing environments. Self-supervised learning allows for automatic labeling of training data by exploiting correlations between input signals to make the vision prediction scheme more autonomous [36–39]. For example, Wellhausen *et al.* [40] extracted friction features from the footprints of a legged robot and the force-torque signal felt by the robot’s feet and meanwhile collected colorful images along the robot’s path so as to form a mapping between vision and touch. However, these attempts still require robots to collect a large amount of data in advance. Once new situations are encountered in the future, they may lack the ability to make proper decisions [41], resulting in various kinds of failures. For example, you are not likely to walk across the lake in advance to figure out whether it is passable, right? Without sufficient data, how do you extract effective features in a dynamically changing environment? Autonomously perceiving the physical characteristics of the environment does not seem to be a good solution.

**Figure 1.**
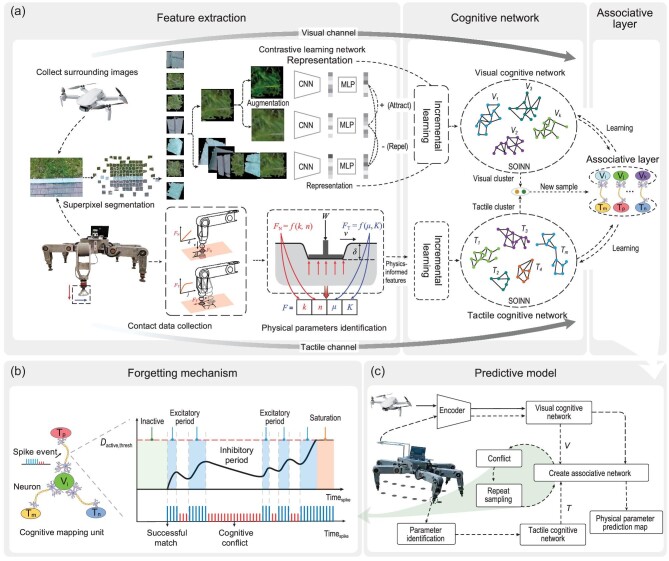
Physical characteristics of the learning framework. (a) Framework for learning the cognitive network and the associative layer. The visual channel contains superpixel segmentation, a contrastive learning network and a visual cognitive network. SLIC is adopted to generate superpixels to offer terrain patches as training samples. Contrastive learning is trained to get an encoder, which can extract latent features of images. E-SOINN is used to train the visual cognitive network, which can cluster visual features in an unsupervised way. The tactile channel contains contact data collection, physical parameter identification and a tactile cognitive network. Through tracking specific foot trajectories, the contact force and displacement are collected in the first part. Identification of physical parameters is achieved using contact models to extract tactile features of terrains. Then, a tactile cognitive network can be created like the visual cognitive network. Finally, the associative layer is created, and the details are shown in (b and c). (b) Forgetting mechanism of modal mapping, which is designed to adjust the connect intensity of mapping pairs (between visual clusters and tactile clusters). It is used to solve cognitive conflicts. (c) Framework for updating the associative layer and predicting physical characteristics from vision, where the solid lines represent the offline training process, and the dashed lines represent the online training and prediction processes.

In this paper, inspired by the animal cognition process, we present a framework for legged robots to perceive physical characteristics of the environment autonomously, as shown in Fig. 1. The tactile perception and the visual perception work independently to build a corresponding feature space and later they are associated together to update the physical cognition of the environment in this framework. On the tactile perception side, features consisting of softness and friction parameters are extracted using foot-terrain interaction models. Specifically, the robot executes a predefined foot trajectory to collect contact force and displacement along normal and tangential directions; then, a unified contact model is utilized to identify terrain parameters (representing softness and the friction level of the terrain) by nonlinear optimization methods. On the visual perception side, an unsupervised feature extraction method is designed: first, the surrounding images collected by an unmanned aerial vehicle (UAV) are segmented into patches as training data by a simple linear iterative clustering (SLIC) method, which can help each patch containing a single type of terrain as much as possible; similar terrain patches are used as positive samples and different terrain blocks are treated as negative ones for training, making positive samples close together while negative ones far away in the feature space. After obtaining tactile and visual features, an enhanced self-organizing incremental neural network (E-SOINN) is adopted to build cognitive networks and cluster all features. Compared with K-means, E-SOINN does not rely on the assumption of the known number of terrain classes and can learn in an incremental manner. After that, clustering results are used as pseudo-labels of image patches to segment terrain images. Finally, an associative layer using spiking neural networks is designed to connect visual and tactile labels, and the activation intensity between them is controlled by trigger spike events, where results matching old knowledge are treated as positive activation events while cognitive conflict situations are regarded as negative activation events. Updating the activation intensity of mapping pairs (a pair is composed of a visual cluster and a tactile cluster) helps the robot deal with cognitive conflicts and renew its physical cognition. The associative layer can also be used to predict physical characteristics through vision, helping legged robots to avoid non-geometric obstacles.

In this work, there are several main contributions beyond state of the art in robot perception. (i) An unsupervised learning framework is proposed to perceive physical characteristics online and incrementally, without the need of traversing a similar environment in advance. (ii) The learning framework allows robots to handle cognitive conflicts, which is, to the best of our knowledge, the first attempt in the field of terrain perception. (iii) A novel unsupervised terrain segmentation method is proposed. Visual features are extracted through contrastive learning and are clustered by E-SOINN to label the image patches cropped by SLIC, forming an unsupervised segmentation framework. (iv) Physical parameters representing the friction and softness of terrains are estimated based on the unified foot-terrain contact models proposed, which can distinguish different terrains effectively. (v) The proposed method is extensively evaluated using a hexapod robot, including indoor and outdoor navigation tasks. It is worth mentioning that our method can be deployed on any legged robots, but is verified only on a hexapod robot. Compared with other works [36,40], one major difference is that we adopt the UAV to collect surrounding images around the robot on the ground; such treatment helps the land robot collect adequate images of the environment, including dangerous terrains, in an efficient and safe way. The reported results include quality tests of visual and tactile features, constructions of cognitive networks and associative layers, visual predictions of physical characteristics, verification of conflict-handling solutions in multi-terrain environments and application in robot navigation.

## RESULTS

Both indoor and outdoor experiments have been carried out. ELSpider (see Fig. S5 within the online supplementary material), an electric-drive heavy-duty hexapod robot (1.9 m × 2.1 m × 0.5 m, 330 kg) developed at the Harbin Institute of Technology, is used for our experiments. Each leg of ELSpider has three degrees of freedom and is equipped with a circular flat foot. Six-axis force-torque sensors are mounted at the connection between the feet and legs to measure normal and tangential forces. The sensor can detect forces of up to 330 N along the *x* and *y* axes and 990 N along the *z* axis, and detect up to 30 Nm of torque at a time. Depth cameras (D435i) are installed on the robot to detect environmental visual information. A tracking camera (Intel T265) is used to track the robot’s pose, and a router with a bandwidth of 3000 Mbps is installed for data communication. In addition, we use the DJI MINI2 UAV for remote visual perception of the environment (the UAV will fly at a low altitude around the robot, and collect images around it), with the data transmitted to a high-performance computer mounted on ELSpider. Note that the camera setting, such as the height and camera angle, has a significant impact on the prediction accuracy (see Section 6 within the online supplementary material); so the camera setting of the UAV is similar to that of the hexapod robot in our experiments. When the robot travels to a new environment, the computer on ELSpider will train a contrastive learning network in advance.

### Outdoor experiments

#### Visual cognitive network

**Figure 2.**
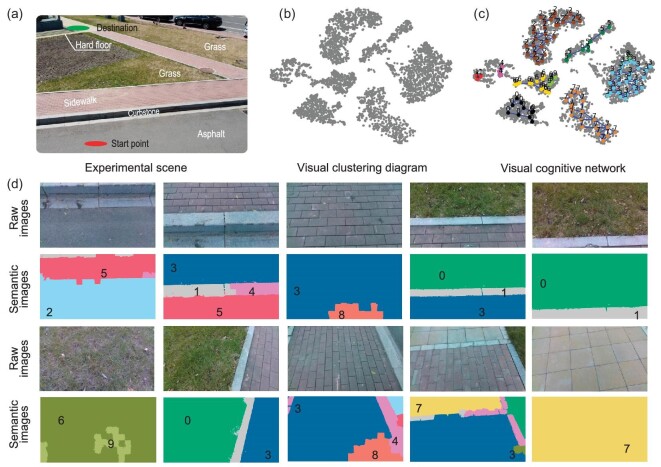
The unsupervised segmentation results. (a) Experimental scene in a realistic outdoor environment with different terrains, including asphalt, sidewalk, curbstone, grass and hard floor. (b) Visualization of visual features using t-SNE. (c) Visual cognitive network created by E-SOINN. It divides visual features into 10 clusters. (d) Results of the unsupervised segmentation, where the second and fourth rows of images correspond to the segmentation results of the first and third rows of raw images.

Figure 2(a) depicts the scene of the outdoor experiment with five types of terrains, including asphalt, grass, curbstone, sidewalk and hard floor. In the first place, the UAV, controlled by a human operator, flies around ELSpider to collect images. The images are transferred to a computer embedded in ELSpider, where they are cropped into patches with 10 000 pixels by SLIC, and subsequently resized as standard rectangle patches (100 × 100) as final input samples. We use these patches to train a contrastive learning network; see Fig. 1(a). For the training, we use the Adam optimizer with a learning rate of 1*e* × 3 and a batch size of 256 for 50 epochs to train the contrastive network. The encoder is ResNet-18, where a multi-layer perceptron (MLP) with one hidden linear layer (512-D input and 512-D output) is used as the projection head; the MLP’s output size is 32-D. See Figure S6 within the online supplementary material for the input and output diagrams. The visualization of features compressed by the network through t-distributed stochastic neighbor embedding (t-SNE) [42] is plotted in Fig. 2(b), showing that the clusters are indeed classified. However, it is hard to evaluate the clustering accuracy without any labels. Therefore, we propose indirectly analyzing the clustering results according to terrain segmentation labeled by unsupervised clustering.

First of all, E-SOINN is adopted to construct a visual cognitive network (VCN; see Fig. 2(c)), which divides all visual features collected by the UAV into 10 categories. Note that the number of categories can be further simplified through human analysis. For example, in Fig. 2(c), class 3 and class 8 can be merged into one cluster. Although E-SOINN with fine-tuned parameters performs well in a specific setting, it may misclassify some different classes into the same class in other scenarios. Thus, a more intuitive solution is to separate the terrain data into more categories than it should have. Interestingly, this treatment will not degrade the performance of our method, as the classified clusters can be combined later using the mapping between tactile and visual data.

After the VCN is trained, the labels predicted by the VCN are used as pseudo-tags for new images. Specifically, given a new image, it is evenly cropped into patches through SLIC. Each patch is compressed by the contrastive network to extract its latent features, which are subsequently fed to the VCN to determine its cluster. In this way, an image can be segmented by splicing with these labeled patches, as shown in Fig. 2(d). By comparison with the corresponding raw image, we can estimate the semantic class for each cluster: cluster 2 corresponds to asphalt; clusters 3 and 8 belong to the sidewalk; cluster 7 is the hard floor; clusters 0, 6 and 9 correspond to grass; clusters 1, 4 and 5 represent curbstone. From the semantic images in Fig. 2(d), we can see that those different terrains are distinguished properly, which satisfies the need for robot navigation.

In order to evaluate the clustering accuracy, we have annotated the images collected by the UAV. The following metric is designed to evaluate the accuracy:


(1) \begin{eqnarray*} \text{clusterAcc}=\sum _{i=1}^N \frac{\rm{MaxSameN}(C_i)}{\rm{Num}(C_i)} / N. \end{eqnarray*}

Here *N* represents the number of clusters, $\rm{Num}(C_i)$ denotes the number of samples belonging to cluster *C_i_* (clustering results of E-SOINN), $\rm{MaxSameN}(C_i)$ is the maximum number of samples that belong to *C_i_* and have the same labels as those annotated by humans. This indicator measures the clustering degree of samples in each cluster—higher values mean better clustering results. By adjusting the parameter of E-SOINN, we can get different groups of clusters. We performed several tests to verify the impact of the number of clusters on the clustering accuracy. As shown in Table S7 within the online supplementary material, the clustering accuracy is above 94%, with the accuracy increasing as the number of clusters increases, i.e. using more clusters can better aggregate samples of the same category. However, dividing one terrain into multiple categories will not affect the effect of our method, because they can be reunited again through the mapping process between tactile and visual data. Therefore, satisfactory clustering accuracy verifies the success of the unsupervised visual feature clustering method.

#### Tactile cognitive network

*Verification of tactile feature extraction.* The tactile feature plays a pivotal role when training the tactile cognitive network (TCN), so here we first verify the effectiveness of the proposed feature extraction solution. In order to extract tactile features sufficiently, we let the robot execute a predefined reference trajectory and collect the corresponding force and displacement along normal and tangential directions. Subsequently, a unified contact model is adopted to identify terrain parameters by nonlinear optimization methods. The design of the reference trajectory and the extraction of tactile features are discussed in the Methods section.

**Figure 3.**
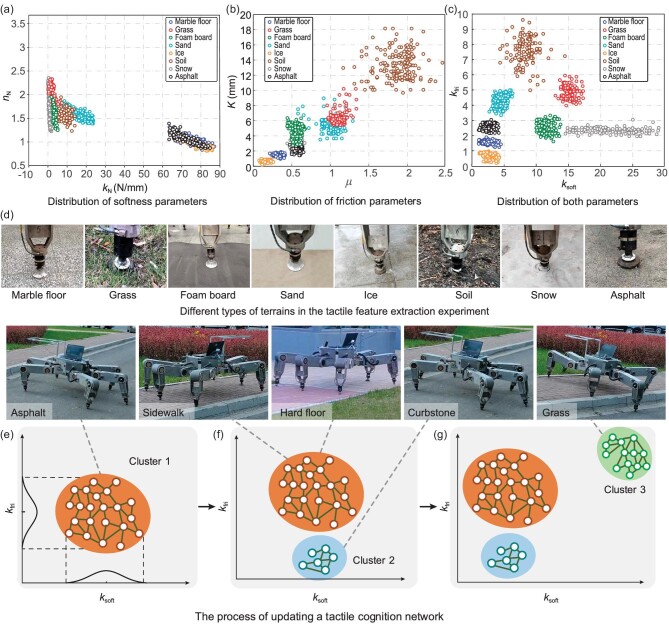
Results of tactile feature verification experiments. (a–c) Visualization of tactile features. Here *k* is the equivalent rigidity of the terrain and *n* represents the exponential coefficient of the terrain’s deformation; μ denotes the frictional coefficient of the terrain and *K* (*m*) is the shearing deformation modulus of the terrain; *k*_soft_ and *k*_fri_ represent the softness and friction parameters of the terrain, respectively. (d) Robot touches different terrains to extract tactile features. (e–g) Process of creating a TCN: asphalt, sidewalk and hard floor are clustered as one type, and curbstone and grass are another two types.

We carried out experiments on eight typical terrains, including marble floor, grass, foam board, sand, ice, soil, snow and asphalt. The extracted softness parameters and friction parameters in various terrains are illustrated in Fig. 3(a) and (b). Interestingly, with these two groups of parameters, all terrains can be classified properly, (see Fig. 3(c)), although some terrains are indistinguishable using a single group of parameters. For example, the softness parameters of asphalt, ice and marble floors are similar, but their fiction parameters are significantly different. The friction parameters of foam board and snow are largely overlapped, but they can still be classified using the softness parameters (although they are very close to each other). Thus, using both the softness and friction parameters as tactile features is advantageous for terrain classification.

We adopted the same metric as that of the visual-feature verification part to verify the clustering accuracy of tactile data. The clustering accuracy results are shown in Table S8 within the online supplementary material. When the number of clusters is larger than the real category number (8), the algorithm can provide higher clustering precision (over 97%). By contrast, the clustering accuracy decreases when the number of clusters decreases.

*Tactile cognitive network.* We now discuss the training of the TCN in an outdoor setting. The scenario is shown in Fig. 2(a), where the robot walks from the starting point (marked by the red ellipse) to the destination (marked by the green ellipse). When the robot works on the asphalt, the first cluster is created in the TCN (see Fig. 3(e)–(g)), representing hard terrains with large friction. When the robot touches the curbstone, the second cluster is created, as the curbstone is more slippery than asphalt. However, as the physical characteristics (i.e. friction and softness) of the sidewalk are similar to that of asphalt, sidewalk is recognized as the same cluster as asphalt. Once the robot touches the grass, a third cluster is created since the perceived tactile features are quite different from those of previous terrains. After that, the robot walks on the sidewalk until it reaches the hard floor near the destination. Because the physical characteristics of the hard floor are similar to those of asphalt, it is classified into the first cluster. Thus, in this navigation task, the TCN network can be created in an incremental way.

*Tactile parameter estimation from clusters.* As shown in Fig. 3(e), each tactile cluster represents a kind of terrain with similar tactile parameters. However, there is still some difference between samples in the cluster. In order to estimate the tactile parameters from clusters, they can be modeled as two norm distributions to present the softness and friction parameters. In this way, once the tactile cluster is predicted from vision, the tactile parameters can be estimated through the distribution.

#### Updating the associative layer

The associative layer aims to build a mapping from visual clusters to tactile clusters. In the process of building the TCN, the depth camera (D435i) will also perceive the environment simultaneously. The captured images will be sent to the contrastive network to extract the corresponding visual features. Whenever a new tactile cluster *T_j_* is added, we can combine it with the corresponding visual cluster *V_i_*, and then update the associative layer (which can be interpreted as a mapping table) in the form of *node* = {*V_i_, T_j_, n*_a_, *n*_c_, γ}, where the indices of the new tactile cluster and visual cluster are respectively *i* and *j, n_a_* denotes the frequency of matched pairs, *n_c_* denotes the frequency of conflicted pairs and γ > 0 represents the activation intensity, which is modeled using a spiking neuron (see Fig. 1(b)). When γ > 0.85, the node is fully activated. By contrast, if γ ≤ 0.15, the node will be deleted. The variation rate of γ is modulated by an additional parameter *K*_LIF_, which is set as 0.5 in our experiment. The associative layer obtained in the navigation task (Fig. 2(a)) is reported in Table S3 within the online supplementary material, where different visual classes are associated with the same tactile class if they exhibit similar mechanical properties.

#### Predicting tactile features

In the navigation task (see Fig. 2(a)), we first train the contrastive learning network using images collected by the UAV. Once the contrastive learning network is ready, we can segment perceived images in the navigation task into different visual clusters online, which can be subsequently used to predict the corresponding tactile clusters via the associative layer. Note that the associative layer is also generated online.

**Figure 4.**
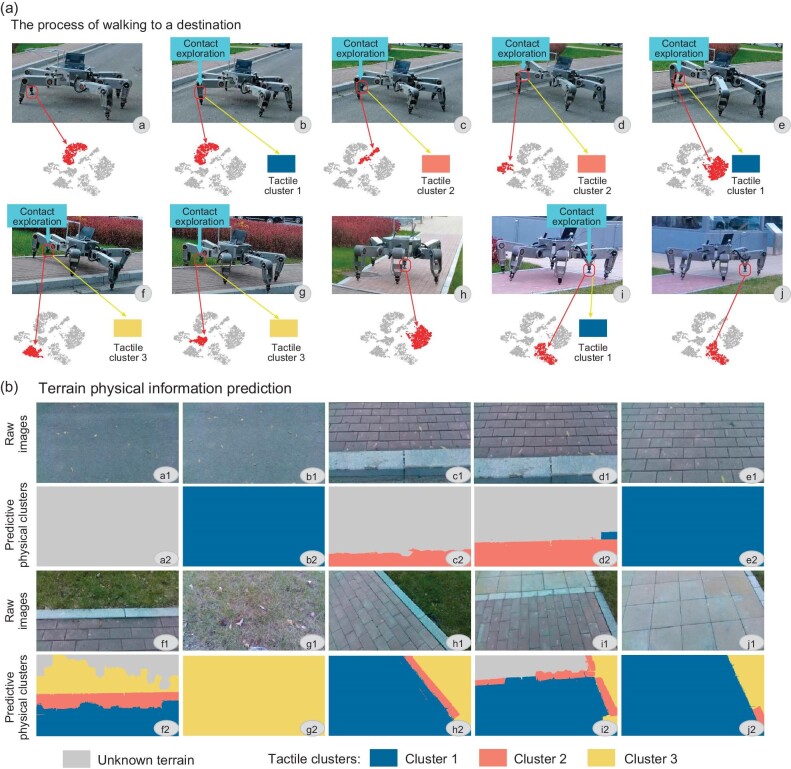
Prediction of the physical characteristics. (a) Walking process of the hexapod robot. (b) Physical prediction map of the raw images, where gray denotes unknown terrains, blue represents terrains that are rigid and with sufficient friction, yellow represents soft terrains and pink represents slippery terrains.

Figure 4 shows the prediction of tactile features in the navigation task. At the very beginning (panel a of Fig. 4(a)), with the image patch containing the foothold of the front foot (bounded by the red box), the corresponding visual cluster can be predicted using the VCN. Since the initial associative layer is empty, the corresponding tactile cluster cannot be predicted (i.e. unknown terrains); see the gray image in panel a2 of Fig. 4(b). However, once tactile information is collected through the front foot interacting with the terrain (panel b of Fig. 4(a)), a new tactile cluster (i.e. cluster 1) will be created. In this case, the associative layer containing the relationship between the current visual cluster and tactile cluster is established, which can be used to predict tactile clusters from images. As the entire raw image in panel b1 of Fig. 4(b) is purely asphalt, all patches inside this image correspond to the same tactile cluster, i.e. cluster 1; see panel b2 of Fig. 4(b). In panel c1 of Fig. 4(b), the image patch containing the foothold of the front foot is classified as a different visual cluster, implying that a new terrain appears; hence, contact exploration is applied. After a new tactile cluster (i.e. tactile cluster 2) is added, the associative layer will be updated accordingly, which can predict the corresponding tactile clusters for the entire image; see panel c2 of Fig. 4(b), which includes cluster 2. Similarly, in panels d1–j1 of Fig. 4(b), the robot updates the associative layer with new visual clusters and the corresponding tactile clusters until it reaches the destination. The final associative layer builds a mapping between eight types of visual clusters and three types of tactile clusters. Note that visual clusters 8 and 9 are not encountered during the whole navigation task; thus, these clusters are not involved in the final associative layer. Once the tactile cluster is predicted from vision, the tactile parameters can be estimated through the parameter distribution of the cluster in the tactile cognition network (see Fig. 3(e)). The process of updating the associative layer is consistent with the way that animals perceive and understand a new environment. As long as the associative relationship is known, animals can instantly estimate tactile features according to their visual observation in new scenarios.

#### Navigation application

In order to apply the prediction results of environmental physical information to robot navigation, we project the predictive tactile clusters onto an elevation map, and construct a physical map of the environment using the open-source elevation mapping package [43]. The entire procedure of building the physical model includes converting raw images into tactile segmentation images; transforming depth images and tactile images into semantic point clouds in the coordinate of the depth camera (see D435i in Fig. S5 within the online supplementary material) according to the camera model; estimating the pose of the depth camera with respect to the world frame using the tracking camera (see T265 in Fig. S5 within the online supplementary material); thus, the semantic point clouds can be projected to the elevation map. In order to mitigate the effect of location drift arising from pose estimation, the tracking camera is combined with a legged odometer to obtain a more stable pose estimator.

Figure S7 within the online supplementary material shows the constructed physical map in the navigation task, where the blue area represents the terrain that is rigid and not slippery, the yellow areas represent soft terrains and the pink areas represent rigid but slippery terrains. It is worth mentioning that the grass area is viewed as risky terrain due to the heavy weight of the robot, and thus the robot will bypass the grass area after it reaches it. In this way, mapping the predicted tactile clusters into elevation maps helps robots navigate safely from the starting point to the destination, avoiding non-geometric obstacles such as grass areas. More details about the navigation experiment can be seen in Demonstration Video S1 within the online supplementary material.

### Indoor experiments

**Figure 5.**
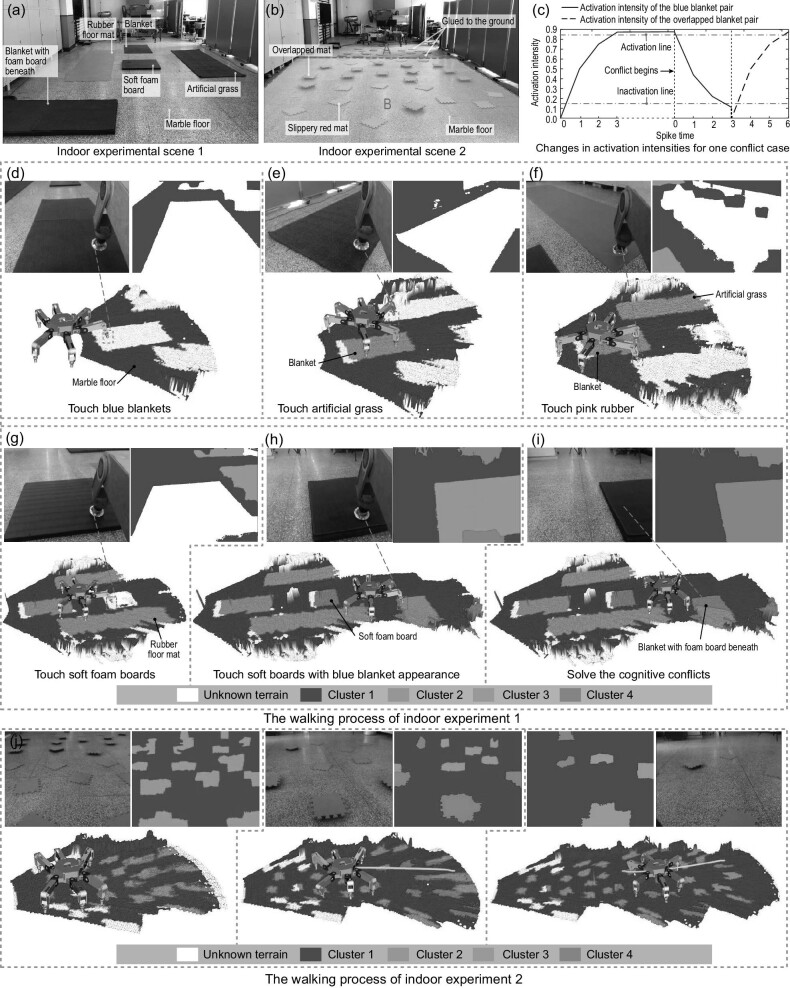
Indoor experiments in two scenes. (a and b) Two indoor scenes. (c) Changes in activation intensities for one conflict case. (d–i) Walking process of the robot in the first indoor experiment, where the upper image in each figure is the raw image and its predictive physical map, and the lower image is its navigation screenshot from rviz (the visualization tool of ROS). (j) Walking process of the second indoor experiment.

Varying terrain environments call for adaptivity of robots, and we carry out indoor experiments to verify the ability of the proposed method to solve cognitive conflicts. indoor scene 1, as shown in Fig. 5(a), includes a marble floor, a blue blanket, an artificial grass area, a soft foam board, a pink rubber floor mat and an overlapped terrain with a blanket terrain over a foam board. The navigation task in scene 1 is illustrated in Fig. 5(d)–(i), where the procedure of building the associative layer is similar to the outdoor experiment (Fig. 4) at its early stage until the robot touches an overlapped terrain (i.e. a blue blanket terrain placed over a foam board). However, the previous blanket terrain seen in Fig. 5(d) was relatively hard and with high friction, but the estimated softness parameter of the overlapped terrain in Fig. 5(h) is unexpectedly small, corresponding to the same tactile cluster as the soft foam board. In order to address the cognitive conflict, the robot continuously touches the overlapped terrain a few times, then updates the spiking neuron in the associative layer. Specifically, as shown in Fig. 5(c), for spike times from 0 to 6, the activation intensity between the visual cluster of the blue blanket and the tactile cluster of the soft foam board rises (when the activation intensity γ > 0.85, the pair is fully activated), while the activation intensity between the visual cluster and the tactile cluster associated with the blanket decreases until γ ≤ 0.15. The predicted tactile map is shown in Fig. 5(i), where the color corresponding to the observed overlapped terrain changes from green to red. Note that a part of the overlapped terrain is still depicted by the green color, implying that this part has not been observed. Therefore, an updated associative layer without cognitive conflicts is obtained. Since the tactile features of the blanket and the rubber floor mat are similar, both terrains are united into one tactile category; see the green areas in Fig. 5(d)–(i); the marble floor, which has high stiffness and low friction, is highlighted blue; the softer and more slippery grass is highlighted orange; finally, the very soft foam board is highlighted pink. All terrain parameters are summarized in Table S1 within the online supplementary material.

The indoor experiment in scene 2 also shows the conflict-solving ability of our method. Indoor scene 2 shown in Fig. 5(b) is classified into two regions. In region A, there are three types of terrain: a marble floor, blue mats and red mats, with the mats glued to the floor. In region B, apart from the marble floor, we design two kinds of terrain: blue mats placed over soft foam boards, where the boards are glued to the floor, and slippery red mats (without glue). From Fig. 5(j), we can see that the hexapod robot is able to distinguish terrains with similar appearances but different physical parameters. In this experiment, once the robot recognizes a dangerous area (i.e. the overlapped and slippery mats), it can avoid placing footholds within the dangerous area. More details can be found in Demonstration Video S3 within the online supplementary material.

### Comparison experiments

**Figure 6.**
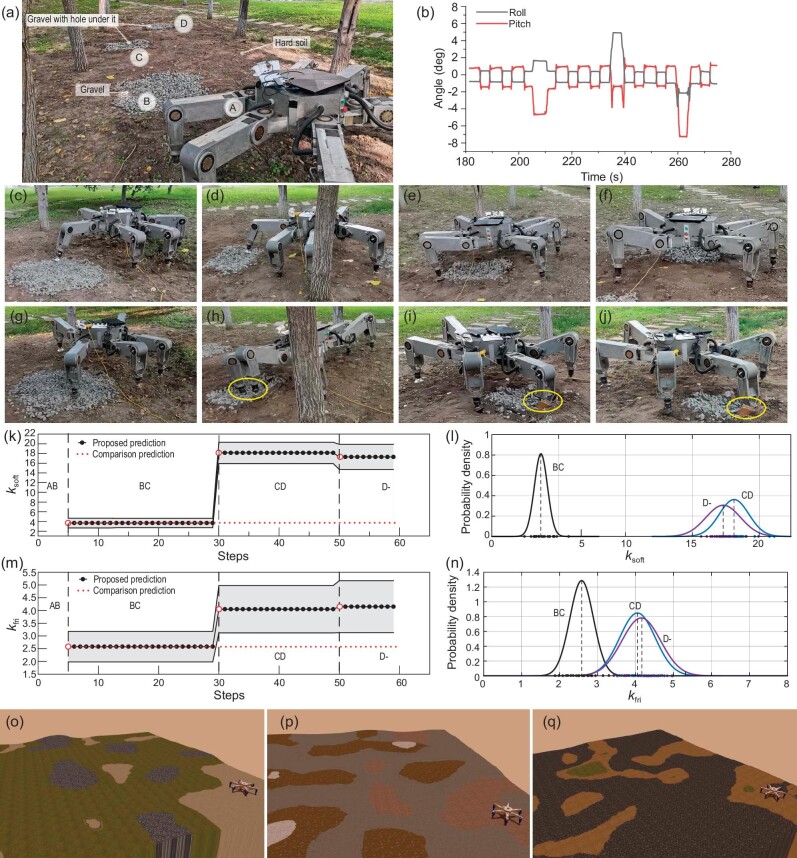
Comparison experiments. (a) Experimental scene. (b) Roll and pitch angles of the robot during the walking process using the comparison method. (c–f) Navigation process using the proposed method. (g–j) Navigation process using the comparison method, where yellow circles show severe subsidence of the robot. (k–n) Physical parameter prediction results. (o–q) Comparison simulation environments.

We performed a comparison between our solution and a self-supervised method [39,40]. Unlike the self-supervised method that requires the collection of hundreds of labeled training data in the form of image patches and their corresponding tactile parameters in advance, our method does not need such labeled training data since it creates and updates a TCN online and thus allows for predicting tactile features using visual observation. In this experiment, the Elspider tries to traverse an unknown environment (see Fig. 6), where there are three types of terrain, including hard soil, gravel and gravel with a hole under it. For the proposed method, the robot touches the gravel terrain in Fig. 6(d), finding that the physical parameter is much softer than that of the gravel in Fig. 6(c), so the robot avoids walking on the gravel area and traverses the scene safely (see Fig. 6(e) and (f)). By contrast, the robot using the self-supervised perception considers the gravel in Fig. 6(h) as a safe terrain even after severe subsidence, and later continues to walk through this terrain (see Fig. 6(i) and (j)) since the physical predictive model is unchanged. However, as depicted in Fig. 6(b), the robot’s posture will change dramatically, which may lead to the robot capsizing.

The gravel terrain has two different physical properties during the walking process: one is solid and slippery, while the other is extremely soft and with greater friction (gravel with a hole under it). Figure 6(k) and (m) show the physical parameter prediction of gravel along the motion of the robot from point A to point D in Fig. 6(a). During the AB phases, the gravel terrain is not predicted because the mapping relationship between the vision and tactile data of gravel is not established. Once the robot touches the gravel at point B, there are predictions of the gravel as normal distributions (*k*_soft_ ∼ *N*(3.67, 0.49), *k*_fri_ ∼ *N*(2.58, 0.31)). However, when the robot touches the gravel with a hole under it at point C, because of the predictive tactile cluster changes in the associative layer, the prediction parameter distributions changed (*k*_soft_ ∼ *N*(18.12, 1.10), *k*_fri_ ∼ *N*(4.05, 0.47)). After that, when the robot touches point D, the prediction tactile cluster does not change but adds a new tactile node in the TCN, leading to an update of the prediction parameter distributions (*k*_soft_ ∼ *N*(17.13, 1.30), *k*_fri_ ∼ *N*(4.15, 0.51)).

Regarding the parameter prediction accuracy, the hexapod robot is controlled to collect the tactile data (each terrain contains 20 samples) as the baseline to be compared with the prediction parameters. The baseline parameters are shown in Fig. 6(l) and (n), represented by dots on the abscissa. The black dots are the parameters of the gravel at point B, while the blue and purple dots represent the parameters of the gravel at points C and D. If the average parameter of each distribution is taken as our predictive physical parameter, the prediction accuracy of tactile parameters is calculated as


(2) \begin{eqnarray*} \text{accuracy}=1-\frac{\sum _{i=1}^N|s_i-p| / N}{p}, \end{eqnarray*}

where *s_i_* is the parameter of sample *i, p* is the prediction parameter and *N* is the number of samples. The prediction accuracy is shown in Table S9 within the online supplementary material. It can be seen that the prediction accuracy of our method is around 90%, and the accuracy is negatively correlated with the variance of the predictive distribution. By contrast, when the terrain’s physical property changes, the prediction using the comparison method differs significantly from the baseline.

To quantify the advantages of our method over the comparison methods in robot navigation, we generated 10 different terrain environments to compare the navigation results using the two prediction methods in the simulation, as shown in Fig. 6(o)–(q). A simulator named MarsSim [44] developed upon the ROS/Gazebo platform that supports both physical and visual realistic simulations is adopted as our simulator. The self-supervised method requires the collection of labeled training data in the form of image patches and their corresponding tactile parameters (around 3000 samples for an environment) in an environment where the physical parameters of the terrain do not change to train the prediction model, while the proposed method only needs to collect environmental images quickly in advance and update its prediction model online. For each simulation environment, the mechanical parameters of a terrain with a similar appearance change in order to imitate challenging environments, and the robot is required to move to two goals with the same starting point using the prediction model trained by different methods. During the movement, the number of severe slips (slip over 3 cm for a foot) and jolt cases (roll or pitch angle over 5°) is registered. Meanwhile, the task completion is recorded. The comparison simulation results are shown in Table S10 within the online supplementary material. We can see that the robot using the proposed method performs all tasks while avoiding severe bumps and slips due to the advantage of adjusting the prediction model of physical characteristics along with the changing environments. However, if using the prediction model trained by the data collected in advance, the robot cannot predict the physical characteristics accurately when those of the terrain change. Because of overtrust in previous experience, the robot fails to adjust its motion in time, which in turn results in substantial sinking and slipping. Admittedly, prudent exercise consumes more time to reach the goal, but the strategy is safer and more reliable in meeting the complex environment. More details can be found in Video 5 within the online supplementary material.

## DISCUSSION

This paper introduced a novel method for legged robots to autonomously learn the physical characteristics of an environment, which is the first step to realizing learning in an autonomous, real-time and incremental way. Compared with previous studies, the proposed method is also the first to solve the cognitive conflict problem. By introducing the forgetting mechanism, the robot can continuously update the prediction model of physical characteristics in dynamic environments, endowing the robot system with higher adaptability.

In terms of the visual model, the proposed method builds a terrain segmentation model without any human labels and classifies terrains into proper categories. In terms of the tactile model, the proposed features consisting of the friction and softness parameters can distinguish different terrains efficiently. By constructing the E-SOINN network, a TCN is constructed incrementally so that the robot can continuously collect tactile data by interacting with terrains. Finally, the visual-tactile associative layer can predict the physical characteristics of terrains without touching them, helping the robot to navigate safely. More importantly, the proposed method can resolve cognitive conflicts and update old cognition in a dynamic environment that greatly improves the robot’s adaptability compared to other methods.

This work still has some limitations. First, the UAV was controlled by a user to collect surrounding images of the legged robot, which may limit its applications, e.g. the human operation is unrealistic in planetary exploration tasks. Second, in order to better train the contrastive learning network, the UAV needs to collect sufficient images for each terrain; in this case, the imbalanced samples may hinder the training of the network. Third, the training time of the vision cognitive network is 2–3 hours, which may prohibit its deployment when the environment changes dramatically and rapidly. For example, when the robot is operating on an urgent task but the weather changes suddenly, the robot should retrain the network with new images quickly.

This paper covers a wide range of areas, which could inspire research in many fields of robotics. In the field of multi-modal perception, the combination of autonomous cognitive techniques and mobile robotics can often achieve much better results than traditional technologies, which has been a research focus recently. In the context of robot navigation, this paper proposes to characterize the physical information of the environment (including non-geometric obstacles) using a contact mechanics model so that safe navigation of the robot is ensured. From the perspective of machine cognition, the solutions to represent, summarize and forget knowledge, as well as resolve cognitive conflicts, are necessary for general artificial intelligence. For multi-robot collaboration, we use remote UAVs to enlarge the perception domain of legged robots, which verifies the effectiveness of such collaboration and could further boost research interests in this direction.

## METHODS

In this section we cover four main parts: visual feature extraction, tactile feature extraction, cognitive network construction and associative layer construction.

### Visual feature extraction

Manually designed features or the supervised learning method cannot adapt to the outdoor environment with changing conditions. The contrastive learning method [45] is adopted to extract visual features from different terrains. The surrounding images collected by a UAV are segmented into patches as training data by a SLIC method, which can help each patch containing a single type of terrain as much as possible. Similar terrain patches are used as positive samples and different terrain blocks are treated as negative ones for training, making positive samples close together while negative ones far away in the feature space. The encoder trained by the contrastive learning method is adopted as the feature extractor.

### Tactile feature extraction

When a legged robot is walking on the ground, its feet interact with the ground, where the contact forces are usually decoupled into normal and tangential forces. Following our previous work [22], the normal and tangential contact models between different types of feet and different terrains are unified. Furthermore, the single parameters characterizing the softness and friction of terrains are proposed as tactile features. In order to identify ground parameters, we let the robot execute predefined motion trajectories consisting of normal press and tangential rub. The collected force and feet displacement profiles are used to estimate the softness and friction degree of the terrain, respectively (see Fig. S4 within the online supplementary material).

### Cognitive network

The cognitive layer is constructed using E-SOINN [46]—an improved version of SOINN [47]. E-SOINN is a competitive learning-based neural network with only two layers, which can perform unsupervised online clustering of dynamic input data without labels, represented by topological structures. The incremental nature of E-SOINN allows it to discover and learn new patterns that appear in the data stream without affecting the previous learning. Another key feature of E-SOINN is that it does not need a prior assumption about the cluster number, thus making it more suitable for our problem. As new tactile and visual features are collected, E-SOINN updates the network and clusters similar terrains incrementally.

### Associative layer

The associative layer consists of the mapping pairs *P_k_*, which include two clusters and three scalars, *P_k_* = {*V_i_, T_j_, n*_a_, *n*_c_, γ}, where *V_i_* represents the *i*th visual cluster of the terrain in the VCN, *T_j_* represents the *j*th tactile cluster of the TCN, *n*_a_ denotes the number of matched pairs, *n*_c_ denotes the frequency of conflicting pairs and γ ≥ 0 denotes the activation intensity of the pair. A spiking neural network [48] is used to connect the pairs, where the activation of a neuron is triggered by discrete spikes. Through the leaky-integrate-and-fire (LIF) model [49], the activation intensities of the pairs change adaptively, making it capable of forgetting and dynamic updating.

More details about the methods can be found in Section S1 within the online supplementary material.

## Supplementary Material

nwad045_Supplemental_Files
