# Supplementary material for: Learning physical characteristics like animals for legged robots
Source: Natl Sci Rev. 2023 Feb 22;10(5):nwad045. doi: 10.1093/nsr/nwad045 (PMC10089589; doi:10.1093/nsr/nwad045)
Supplement: nwad045_Supplemental_Files [file nwad045_supplemental_files.zip › Supplementary_Materials.pdf]

Learning physical characteristics like animals for legged robots (Supplementary Materials)

Peng Xu[1,+], Liang Ding[1,+,*], Zhengyang Li[1], Huaiguang Yang[1], Zhikai Wang[1], Haibo Gao[1], Ruyi Zhou[1], Yang Su[1], Zongquan Deng[1], and Yanlong Huang[2]

[1]Key Laboratory of Robotics and Systems, Harbin Institute of Technology, Harbin 150001, China.
[2]School of Computing, University of Leeds, Leeds LS29JT, UK.
[*]Corresponding Author. Email: liangding@hit.edu.cn
[+]these authors contributed equally to this work

ABSTRACT

Physical characteristics of terrains, such as softness and friction, provide essential information for legged robots to avoid non-geometric obstacles, like mires and slippery stones, in the wild. The perception of such characteristics often relies on tactile perception and vision prediction. Although tactile perception is more accurate, it is limited to close-range use; by contrast, establishing a supervised or self-supervised contactless prediction system using computer vision requires adequate labeled data and lacks the ability to adapt to the dynamic environment. In this paper, we simulate the behavior of animals and propose an unsupervised learning framework for legged robots to learn the physical characteristics of terrains, which is the first report to manage it online, incrementally, and with the ability to solve cognitive conflicts. The proposed scheme allows robots to interact with the environment and adjust their cognition in real time, therefore endowing robots with the adaptation ability. Indoor and outdoor experiments on a hexapod robot are carried out to show that the robot can extract tactile and visual features of terrains to create cognitive networks independently; an associative layer between visual and tactile features is created during the robot's exploration; with the layer, the robot can autonomously generate a physical segmentation model of terrains and solve cognitive conflicts in an ever-changing environment, facilitating its safe navigation.

Supplementary materials

S1. Methods

The methods conclude four main parts: extract tactile features and visual features, establish cognitive networks, and an associative layer. For visual feature extraction, contrastive learning is adopted, which trains an encoder to make latent features of the same type of terrains stay as close as possible and latent features of different terrains as far away as possible. In terms of extracting tactile features, the contact models between terrains and feet are unified first, and then a special leg motion is designed to explore and collect tactile data during the interaction between feet and terrains; finally, tactile parameters are identified. Regarding cognitive networks, E-SOINN is adopted to perform unsupervised clustering of visual and tactile features. Unlike K-means clustering, E-SOINN can cluster incrementally without specifying the number of clusters beforehand. Finally, referring to the structure of human nerve tissues, an associative layer is designed to tackle cognitive conflicts, which is similar to white matter tracts of the human brain to connect different cognitive areas of the brain[1].

Visual Feature Extraction

Manually designed features, such as color and brightness, are widely used by traditional methods to distinguish terrains. However, researchers need to adjust the weight of each feature elaborately for a satisfactory result. Although supervised learning can avoid this problem by feeding enough training data, it takes much effort to make labels, which is impractical for autonomous robots. The success of unsupervised learning in natural language processing in recent years has also provided new ideas for unsupervised image feature extraction, such as contrastive learning work MOCO[2], SimCLR[3], etc, which show promising results in visual representation. Therefore, we attempt to extract terrain features through the contrastive learning method to reduce human participation.

The framework of contrastive learning is simple and effective based on the work of Chen etc.[3]. We adopt it in our work to extract visual features from different terrains. Specifically, this process has five main parts: Image samples creation, data augmentation, encoder, projection head, and loss function. First of all, the problem of contrastive visual embedding needs to be defined.

1) Defination The robot collects images of its surrounding environment remotely through the camera, and sets them as an image set $I = \{\mathbf{x}_1, ..., \mathbf{x}_p\}, \mathbf{x}_i \in \mathbb{R}^d$. The images are fed to contrastive learning network to generate a mapping function $G : \mathbb{R}^d \mapsto \mathbb{R}^a$ $(a << d)$ satisfies

$$s(G(\mathbf{x}), G(\mathbf{x}^+)) >> s(G(\mathbf{x}), G(\mathbf{x}^-)), \tag{1}$$

where $s(\cdot,\cdot)$ measures the similarity of two inputs; G is the encoder in comparative learning, aiming to reduce the dimensionality of the image and obtain its latent feature; $\mathbf{x}^+$ means the positive sample while $\mathbf{x}^-$ represents the negative sample.

2) Image samples creation To obtain image samples that contain as one terrain as possible, the SLIC is adopted to generate patches (i.e., superpixels). In SLIC, we only need to adjust the parameter k, representing the desired number of superpixels, to satisfy the segmentation requirements. For each image with N pixels in color space, we first determine cluster centers $(l_i, a_i, b_i, x_i, y_i)$ using $S = \sqrt{N/k}$ equally sized grids, where (l_i, a_i, b_i) represents the CIELAB color space, (x_i, y_i) is the pixel's coordinate. In order to avoid cluster centers located at the edge of an image, calculating the color distance gradient and adjusting the locations with the lowest gradient is adopted.

The distance between two pixels is defined as

$$\begin{aligned} d_c &= \sqrt{(l_j - l_i)^2 + (a_j - a_i)^2 + (b_j - b_i)^2} \\ d_s &= \sqrt{(x_j - x_i)^2 + (y_j - y_i)^2} \\ D &= \sqrt{\left(\frac{d_c}{N_c}\right)^2 + m^2 \left(\frac{d_s}{N_s}\right)^2}. \end{aligned} \tag{2}$$

where d_c denotes the distance in color space, d_s denotes the XY spatial distance, and D is the combination distance. The parameter m adjusts the weight of color similarity and spatial proximity. Each pixel belongs to the cluster whose center is nearest to the pixel, and finally, the centers of all clusters are updated via K-means until all centers converge. In this way, an image can be cropped into k superpixels, which are later resized as the rectangle image for network training.

3) Data Augmentation Data augmentation is used to enrich data in the field of supervised representation learning and supervised learning[2,4]. We use three augmentation strategies: (1) random cropping, (2) random Gaussian blur, (3)random exposure. Note that images should be re-scaled to the original size after the first operation. Since color is an important factor in distinguishing terrains, random color distortion is not adopted here.

4) Encoder We use ResNet[5] as the encoder $h(\cdot)$ to extract latent features from augmented data samples. Specifically, for an input image $\mathbf{x}_i$, its corresponding representation is $h_i = h(\mathbf{x}_i) = Ave(\text{ResNet}(\mathbf{x}_i))$, where $Ave(\cdot)$ denotes an average pooling (spatial pooling) layer.

5) Projection Head A multilayer perceptron containing only a hidden layer is adopted as the projection head, which can map the resulting latent vector $\mathbf{h}_i$ into the contrastive-loss application space. $\mathbf{z}_i$ is calculated as

$$\mathbf{z}_i = W_2 \sigma(W_1 \mathbf{h}_i), \tag{3}$$

where $\sigma(\cdot)$ denotes the non-linear ReLU activation function, W_1 and W_2 are the weight coefficients of different layers.

6) Loss Function In our work, a mini-batch of N raw images is sampled randomly. For each raw image $\mathbf{x}_i$, we use the augmentation strategies to generate two new images as a pair of positive samples, then we use the $2N$ new images for training. For each pair of positive samples, the remaining $N-1$ pairs of samples ($2N-1$ images in total) are viewed as negative samples.

The similarity of two samples is measured by the cosine similarity applying to their corresponding latent features, i.e.,

$$s(\mathbf{v}, \mathbf{u}) = \frac{\mathbf{v}^T \mathbf{u}}{\|\mathbf{v}\| \, \|\mathbf{u}\|}, \tag{4}$$

where $\mathbf{v}$, $\mathbf{u}$ are latent vectors generated from the encoder. With such similarity metric, the loss function[6] for any pair of positive samples is defined as

$$\ell_{i,j} = -log \frac{\exp(s(\mathbf{z}_i, \mathbf{z}_j)/\tau)}{\sum_{k=1}^{2N} \mathbb{1}_{[k \neq i]} \exp(\text{s}(\mathbf{z}_i, \mathbf{z}_k)/\tau)}, \tag{5}$$

where τ represents the temperature parameter; $\mathbb{1}_{[k \neq i]} \in 0, 1$ denotes an indicator function, and its value is set as 1 if $k \neq i$. The losses across all positive pairs constitute the final loss. Note that both $\ell_{i,j}$ and $\ell_{j,i}$ are computed for each positive pair.

Tactile Feature Extraction

Extracting tactile features based on terrain-foot contact models

When a legged robot is walking on the ground, its feet interact with the ground, where the contact forces are usually decoupled into normal and tangential forces. The normal force represents the support force provided by the ground, which reflects the softness of the terrain indirectly, while the tangential force represents the driving force provided by the ground, which reflects the friction degree of terrains[7]. Establishing contact models can help to measure the softness and friction of terrains.

The foot-terrain contact can be divided into four types: (1) soft feet contact with hard terrains; (2) soft feet contact with deformable terrains; (3) rigid feet contact with deformable terrains; (4) rigid feet contact with hard terrains. The fourth case is often ignored because springs or soft materials are usually added to the end of the feet in order to mitigate damages caused by the hard contact with the robot.

Following our previous work[7], the first three contact models are unified as

$$F_{\text{N}} = k_{\text{N}} \delta^{n_{\text{N}}}, \tag{6}$$

where $0 < \delta < 1$ is the summation of foot and terrain deformation, F_{N} is the normal force, k_{N} and n_{N} respectively denote an equivalent rigidity parameter and an exponential parameter, depending on feet materials and terrain types. When the same feet are used for pressing experiments, the measured data, including deformation and interaction force, is only affected by the terrain properties. In other words, for a well-designed robot, parameters k_{N} and n_{N} are decided by terrains because the shapes and materials of feet are determined already.

We use the softness parameter of terrains to characterize the softness of terrains, i.e.,

$$k_{\text{soft}} = \left(\frac{F'_{\text{N}}}{k_{\text{N}}} \right)^{1/n}, \tag{7}$$

where F'_{N} is the nominal contact force. Note that the larger k_{soft} is, the softer the terrain is and the higher the crossing cost is for the robot. More details on the derivation of equation (7) are provided in the supplementary material S2.

Similarly, tangential contact models in types (1)–(3) are united[7] using

$$F_{\text{T}} = -\text{th}(d, 1.5K) \mu F_{\text{N}}, \tag{8}$$

$$\text{th}(d, K') = \frac{e^{d/K'} - e^{-d/K'}}{e^{d/K'} + e^{-d/K'}}, \tag{9}$$

where F_T denotes tangential force, d represents terrain deformation along the tangential direction, and K is the shearing deformation modulus of the terrain. μ is the frictional coefficient related to terrain properties and the materials used at the foot ends. If the terrain deformation d is larger than $2K$, the tangential force reaches a saturation value of μF_N. When contacting with hard terrains, K becomes small because there are no slippery feet when static friction is switched to dynamic friction.

In the unified shearing model (equation 8), each curve contains an ascending phase and a saturation phase. The value of μ determines the slope of a curve while the saturation point is determined by K. We propose to use a single parameter to represent the friction degree between feet and terrains:

$$k_{\text{fri}} = \omega_1 \cdot \text{atan}\left(\left|\frac{-\mu \cdot \text{th}(3K, 1.5K)\text{F}_\text{N}}{3K}\right|\right) + \omega_2 \cdot \mu \cdot \text{F}_\text{N}. \tag{10}$$

k_{fri} is positively correlated with the friction degree of the ground. The larger the parameter is, the less likely the ground is to be slippery. Since k_{soft} and k_{fri} describe the friction property of terrain, they are taken as feature factors for tactile clustering. More details on the derivation of equation (10) are provided in the supplementary material S2.

Tactile motions inspired by terramechanics

In order to identify ground parameters, we let the robot execute predefined motion trajectories consisting of normal press and tangential rub. The collected force and feet displacement profiles are used to estimate the softness and friction degree of the terrain, respectively (see Fig. S4).

1) Normal Press As shown in Fig. S4A, a normal press motion is applied to the terrain to collect its bearing characteristic curve. This curve, representing the terrain rigidity, shows that the normal force F_N is proportional to the vertical deformation of the terrain. To eliminate the impact of foot velocity, the normal press force is exerted on the terrain slowly and smoothly. We collect the normal force and vertical displacement when the foot touches the ground until the normal force reaches a given threshold.

2) Tangential Rub In order to obtain the relationship between tangential displacement and force, a specific friction motion is designed to move its feet along the terrain surface forward and backward slowly. Specifically, the foot exerts a certain normal force to contact with the terrain, where an admittance control is adopted to ensure the desired contact force. The difference between the expected normal force and the actual force is used as the input of the controller, and the foot position is adjusted by the admittance controller. The control framework takes joint position control as an inner loop and the admittance controller as an outer loop. In this way, the normal contact force between feet and terrains keeps steady. As the foot moves tangentially, the tangential force will increase to a saturated value (see Fig. S4B). Here, the increasing rate and saturated value, determined by terrain types and foot shape, can be used to characterize the friction parameters of the terrain.

Parameter Identification

In the unified model (equation 6) along the normal direction, the softness parameters k_N and n_N are obtained by optimizing

$$\min_{k_N, n_N} \sum_{i=1}^{m} \left(k_N \delta(t_i)^{n_N} - F_N(t_i)\right)^2, \tag{11}$$

where t_i is the i-th sampling time and m is the sampling size.

For the tangential model in (equation 8), the parameters K and μ are determined by optimizing

$$\min_{\mu, K} \sum_{i=1}^{m} \left(-\text{th}\big(x(t_i) - x(0), 1.5K\big)\mu F_N - F_T(t_i)\right)^2, \tag{12}$$

where $x(0)$ represents the initial position of the foot on the terrain, while $x(t_i)$ denotes the foot position at the i-th sampling.

Cognitive Network and Associative Layer

Piaget's cognitive development theory points out that only when babies have perceptual-motor abilities can they start to truly understand things in the environment and form a representation of the original samples of objects[8]. The entire cognitive development of infants involves multiple modalities. When several modalities appear at the same time, the brain will establish internal connections among the modalities. Inspired by the infant's cognitive process, this section constructs a multi-modal cognitive framework for robots. As shown in Fig. 1A in the main paper, the framework includes a VCN and a TCN; both are constructed by E-SOINN, where the associative layer is used to establish the correlation between vision and touch. Through online learning, this method can gradually learn the physical characteristics of the environment and realize the cognitive development of the robot.

1) Cognitive Network The cognitive layer is constructed using E-SOINN[9] – an improved version of SOINN[10]. SOINN is a competitive learning-based neural network with only two layers, which can perform unsupervised online clustering of dynamic input data without labels, represented by topological structures. By contrast, E-SOINN only retains the first layer of SOINN in

structure and removes the intra-class node insertion operation to simplify the training process, making E-SOINN more suitable for real-time incremental learning. The incremental nature of E-SOINN allows it to discover and learn new patterns that appear in the data stream without affecting the previous learning. Compared with unsupervised clustering methods like Gaussian mixture models (GMM) and K-means, another key feature of E-SOINN is that it does not need a prior assumption about the cluster number and thus making it more suitable for our problem.

The trained E-SOINN has a topological structure, where nodes are connected by edges. This topological structure will be updated dynamically when new data inputs are collected. Specifically, for an input vector ε, SOINN finds the nearest node ε_1 (winner) and the second-nearest node ε_2 (second winner) by

$$s_1 = \text{argmin}_{c \in A} \|\varepsilon - \mathbf{W}_c\|, \tag{13}$$

$$s_2 = \text{argmin}_{c \in A \setminus \{s_1\}} \|\varepsilon - \mathbf{W}_c\| \tag{14}$$

where A presents the node set in E-SOINN, and $\mathbf{W}_c$ denotes node c's weight vector. Then it will judge which winner cluster the new input belongs to according to their similarity threshold T_i, which is decided by the maximum distance calculated between node i and its neighbors

$$T_i = \max_{j \in N_i} \left\|\mathbf{W}_i - \mathbf{W}_j\right\|, \tag{15}$$

where N_i is node i's neighbor set, $\mathbf{W}_i$ is the weight vector of node i. Once the distance between winners and the input vector is beyond their thresholds, it will be created as a new node inserted into the network. Otherwise, it will connect with the winner that satisfies the threshold condition with an edge, and meanwhile, the age of all previous edges connected to this winner will increase by "1". Note that if the age of an edge is larger than an age threshold, it will be removed. In this way, the notes can be divided into several clusters. In addition, we use the solutions in the work[9] to find the overlapped areas between clusters and separate them as subclasses in a better way. Finally, after removing noisy nodes according to the probability density of nodes, all notes in E-SOINN will be labeled using the connection relationship. As new tactile and visual features are collected, E-SOINN can update the network and cluster similar terrains incrementally.

After the VCN and TCN are trained, the prediction clustering results can be obtained as their outputs for given visual features and tactile features. Object-oriented programming language can easily create the data structure of a cognitive network, represented by a class containing clusters, their nodes, the connections, node weight, etc.

2) Associative Layer The human brain has three white matter tracts used to connect different areas of the brain and dominate object recognition and understanding functions[1]. In this section, an associative layer is designed to simulate the structure. This structure can learn the associated mapping between the clusters of two cognitive networks, and predict one modality from the other modality according to the established mapping. We now explain the key ingredients of the associative layer separately.

(i) Definition of associative layer In the associative layer, an arbitrary pair P_k comprises two clusters and three scalars, i.e.,

$$P_k = \{V_i, T_j, n_\mathrm{a}, n_\mathrm{c}, \gamma\}, \tag{16}$$

where V_i represents the i-th visual cluster of terrain in the VCN; T_j represents the j-th tactile cluster of the TCN; n_a denotes the number of matched pairs while n_c represents the frequency of conflicted pairs; $\gamma \geq 0$ denotes the activation intensity of the pair.

(ii) Establishing associative layer In Fig. 1A and Fig. 1C in the main paper, we use two channels to establish the entire cognitive framework. For the visual channel, the images which are collected in advance by a UAV flying around the hexapod are cropped as image patches through SLIC. By doing so, each image patch is ensured to contain as one terrain as possible. The patches will be fed to the contrastive network in order to obtain an encoder capable of extracting visual features. With the extracted features, E-SOINN can construct a VCN. Once the VCN is trained, it can be deployed for online visual recognition. For the legged robot, it first captures the image patches of its footholds, which are then sent to the VCN to determine the nearest cluster V_i. On the other hand, the robot will collect tactile data for footholds. Then the tactile features are obtained by identifying the parameters of the foot-terrain contact models, and the features are used to train the TCN in real-time. Subsequently, the tactile clusters T_j corresponding to the tactile features can be found in the TCN.

After both the visual cluster V_i and the tactile cluster T_j are known, they will be used to update the associative pair. To be specific, if V_i does not exist in the associative layer, a new pair is created, and the value of n_a is assigned to 1; if V_i appears in the associative layer and its corresponding tactile cluster in the layer is also T_j, the parameter n_a of the old pairs (V_i, T_j) will increase by "1", implying that the new observation "matches the old cognition"; if V_i appears in the associative layer but its corresponding tactile cluster in the layer is not T_j, the parameter n_c of all old pairs related to V_i will be added by "1", implying that the new pair contradicts the old cognition. Finally, the activation intensity of all pairs related to the visual cluster V_i in the associative layer will be calculated using equation (19), which will be explained later.

(iii) Handling cognitive conflicts A fundamental learning ability of animals is the "ability to handle cognitive conflicts", which allows them to adapt to ever-changing environments. Spiking neural network (SNN)[11] – the third generation of artificial

neural networks, can mimic a biological neural network more closely than the second-generation neural networks[11] and provide an effective tool to resolve the conflict issue. In SNN, the activation of a neuron is triggered by a number of discrete spikes. When the accumulated voltage is greater than a threshold, the neuron is activated. By contrast, if there are no new events transmitted to the neuron, its accumulated voltage will decay to zero. This process makes SNN suitable for designing a forgetting function to balance the old and new cognition.

In a navigation task, it is possible that new terrains that have similar visual clusters as previous experience terrains now have very different physical characteristics. For example, in Fig. 1B in the main paper, a visual category V_i may correspond to several tactile clusters. When a cognitive conflict occurs, the robot will interact with the ground proactively, aiming to identify the reliability of the new pair and update the activation intensity of old pairs in the associative layer. Specifically, for each connection, we construct a spiking neuron to control the activation intensity of the mapping between visual and tactile clusters. When the activation intensity γ is greater than a predefined activation threshold γ_{activate}, the mapping is fully connected; whereas when γ is smaller than a predefined deactivation γ_{forget}, the mapping is deactivated in the associative layer. In this way, cognitive conflicts can be solved by modulating the mapping relationships. Leaky-Integrate-and-Fire (LIF) model[12] is adopted in this study as the spiking neuron model, and the original LIF model is

$$\tau_m \cdot \frac{dV}{dt} = -(V - E_L) + \frac{I}{g_L}, \tag{17}$$

where V denotes membrane potential, I is external input current, E_L represents resting potential, g_L is leak conductance, and τ_m is membrane time constant. We discrete the LIF model as

$$V_{j+1} = V_j + K_{\text{LIF}}(-V_j + I_{\text{spike}}/g_L) + E_L, \tag{18}$$

where K_{LIF} is related to the discrete time step and membrane time constant τ_m. When the resting potential E_L sets as zero, and leak conductance g_L is consolidated into input current I_{spike}, the model can be changed to the following form to adapt to our problem.

$$\gamma_{j+1} = \gamma_j + K_{\text{LIF}}(-\gamma_j + I_{\text{spike}}), \tag{19}$$

where parameter activation intensity γ is changed as the number of visits to visual cognition node V_i increases; the resting potential E_L is assigned to 0, so we ignore this parameter in our model; parameter K_{LIF} control the speed of γ reaching saturation, the larger K_{LIF} is, the more times need to trigger, and vice versa; I_{spike} refers to trigger spike events that change the activation intensity of neurons. Due to the difference of its value, positive excitation or inhibitory excitation can be induced. Specifically, if the cognitive conflict occurs, $I_{\text{spike}} = -1$, which will decrease the activation intensity of pairs related to V_i except the new pair; In contrast, if only a pair can be found in the associative layer, $I_{\text{spike}} = 1$, it will increase the activation intensity of the pair in the associative layer.

(iv) Predicting physical characteristics When the associative layer is built, it will be used to predict physical characteristics of terrains from the perceived vision information. The flow charts connected by dashed lines (see Fig. 1C in the main text) depict such prediction. First, the online collected images by the hexapod robot are cropped into image patches, which will be sent to the encoder (trained by contrastive learning) to extract visual features. After feeding these features to the VCN, the corresponding visual clusters can be found. Finally, the associated layer will predict tactile clusters using visual clusters. Note that each tactile cluster may include several nodes, and each node has a group of physical parameters. We can model the distribution of parameters within each cluster using a norm distribution and use this distribution to represent the predicted physical parameters.

S2. Characterization of Ground Physical Properties

Softness Parameter

Following our previous work[7], the first three contact models are unified as

$$F_{\mathrm{N}} = k_{\mathrm{N}}\delta^{n_{\mathrm{N}}}, \tag{20}$$

where $0 < \delta < 1$ is the summation of foot and terrain deformation, F_{N} is the normal force, k_{N} and n_{N} respectively denote an equivalent rigidity parameter and an exponential parameter, depending on feet materials and terrain types. When using the same feet for the press tests, the variation of the interaction force and the deformation only are attributed to the difference in terrain properties. For well-designed robots, the shapes and materials of feet are determined thus parameters k_{N} and n_{N} are decided by terrains.

In order to compare the influence of parameters k_{N} and n_{N} on the softness of terrains, the bearing characteristic curves under different k_{N} or n_{N} values are drawn as shown in Fig. S1. In Fig. S1A, when n is fixed at 1.5, as the value of k increases, it can be seen that the slope of the curve gradually increases, and the stiffness of the corresponding terrains also rises. In Fig. S1B, when k is fixed, as the value of n changes from 0.8 to 1.8 by 0.05, the slope of curves gradually decreases, and the stiffness of the corresponding terrains also decreases.

It can be seen that the softness of the terrain is determined by both the equivalent stiffness coefficient k_{N} and the exponential coefficient n_{N}. However, there are still some unreasonable situations that suffer from simply combining the two linearly to measure the normal softness of the ground. As shown in Fig. S1B, curve $s_1(k_{\mathrm{N}} = 1\times 10^4, n_{\mathrm{N}} = 1.1)$ and curve $s_2(k_{\mathrm{N}} = 2\times 10^4, n_{\mathrm{N}} = 1.35)$ are the bearing characteristic curves of two different terrains. When the normal force is F'_N, the subsidence corresponding to curve s_2 is smaller than the subsidence corresponding to curve s_1, which represents that the terrain corresponding to s_1 is softer than that corresponding to s_2. By contrast, when the normal force becomes F''_N, the result is completely opposite. Therefore, the softness of terrain is different for robots with various weights. The softness of terrains is influenced by not only the terrain parameters but also the normal force. For example, cardboard may be hard for a lightweight robot, but it's sinkable for a heavy-duty robot. Based on the above analysis, a single parameter of terrains can be designed to characterize the softness of terrains.

$$k_{\mathrm{soft}} = \left(\frac{F'_N}{k}\right)^{1/n} \tag{21}$$

where F'_{N} is the nominal contact force. Note that the larger k_{soft} is, the softer the terrain is and the higher the crossing cost is for the robot.

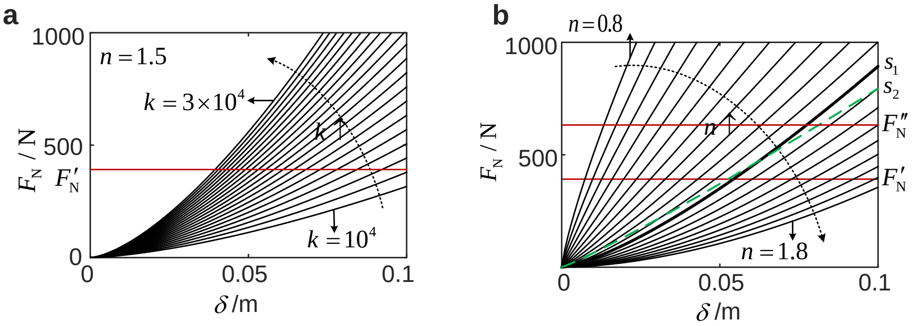

Figure S1. The relationship between normal force and subsidence for different terrain parameters.

Friction Parameter

Similarly, tangential contact models in types (1)–(3) are united[7] using

$$F_{\mathrm{T}} = -\mathrm{th}(d, 1.5K)\mu F_{\mathrm{N}}, \tag{22}$$

$$\mathrm{th}(d, K') = \frac{e^{d/K'} - e^{-d/K'}}{e^{d/K'} + e^{-d/K'}}, \tag{23}$$

where F_{T} denotes tangential force, d represents terrain deformation along the tangential direction, and K is the shearing deformation modulus of the terrain. μ is the frictional coefficient related to terrain properties and the materials of feet. If the

terrain deformation d is larger than $2K$, the absolute value of the horizontal force is μF_{N}. When contacting with hard terrains, K becomes small because there are no slippery feet when static friction is switched to dynamic friction.

The foot-terrain tangential interaction can be jointly measured by two parameters, the maximum tangential force that the terrain can provide and the tangential displacement when the maximum tangential force is reached. As shown in Fig. S2A, when $K = 0.01$ and the value of F_N is constant, as the μ increases from 0.3 to 0.5 by 0.005, the maximum tangential force of the curve increases, which means the driving force that the corresponding terrain can provide to the robot also increases. In Fig. S2B, μ is set to 0.3. As K increases from 0.01 to 0.03, the terrain represented by the shear characteristic curve becomes softer. To achieve the target tangential force, the robot needs to rub in the terrain for a longer displacement. Here, k_{fric} is set to measure the degree of tangential friction of the terrain, which is expressed as follows,

$$k_{\mathrm{fri}} = \omega_1 \cdot \mathrm{atan}\left(\left|\frac{-\mu \cdot \mathrm{th}(3K, 1.5K)\mathrm{F_N}}{3K}\right|\right) + \omega_2 \cdot \mu \cdot \mathrm{F_N}, \tag{24}$$

where the first term of equation (24) ($\omega_1 \cdot \mathrm{atan}\left(\left|\frac{-\mu \cdot \mathrm{th}(3K,1.5K)\mathrm{F_N}}{3K}\right|\right)$) represents the angle between the x-axis and the line connecting the origin and the saturation point; the second term represents the maximal tangential force the terrain can supply (see Fig. S2C). k_{fri} is positively correlated with the friction degree of the ground. The larger the parameter is, the less likely the ground is to be slippery.

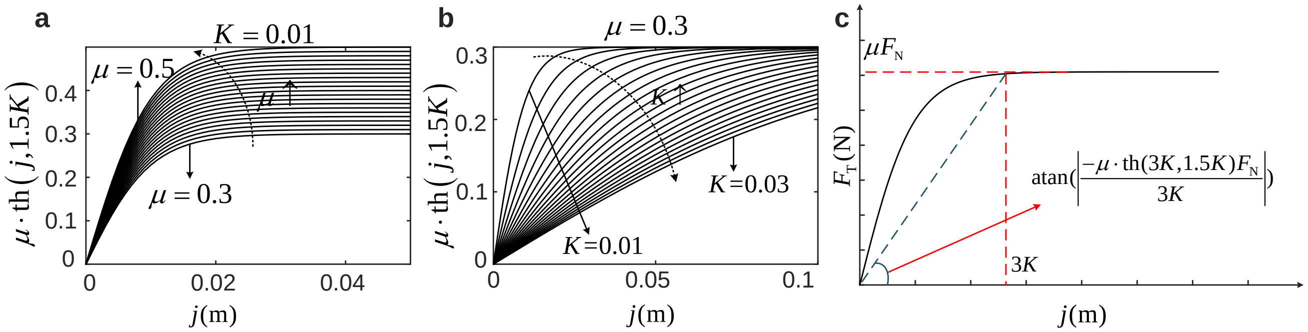

Figure S2. The relationship between tangential force and shear displacement for different terrain parameters.

S2. Hardware System

The experimental system of the legged robot adopts a hierarchical control framework, as shown in Fig. S3, including the following four layers:

1. Hardware layer. This layer mainly includes the hardware composition of the robot system and sensor data collections, including images of the environment, point clouds, and foot force data.

2. Real-time controller layer. This layer receives high-level planning commands and controls positions and forces of joint forces in real-time control algorithms. We adopted the Beckhoff controller in the system.

3. Upper-level computing system. The system runs on the ROS system, receiving constructions from the human-robot interactive interface layer, and all algorithms proposed in this paper run in this layer. It includes the process of setting the whole environmental cognition system by collecting environmental images and contact data. Meanwhile, the planning algorithms are also operating here, which contain the planning of footholds, gaits, and posture of the hexapod robot.

4. Human-computer interactive layer. The locomotion of the legged robot in this paper is not completely autonomous, and the guided path is controlled by the operator. The operator will make decisions by watching the environmental map models and physical information prediction map from the interface.

Remote communication between the Human-robot Interactive Interface and the Upper-level Computing System is via ROS with a communication frequency of 100Hz; the Upper-level Computing System is connected to the Real-time Controller (Beckhoff controller) via EtherCAT with 1000Hz communication frequency; 485 serial communications is used to connect with the motors of ELspider and Real-time Controller with 1000Hz communication frequency.

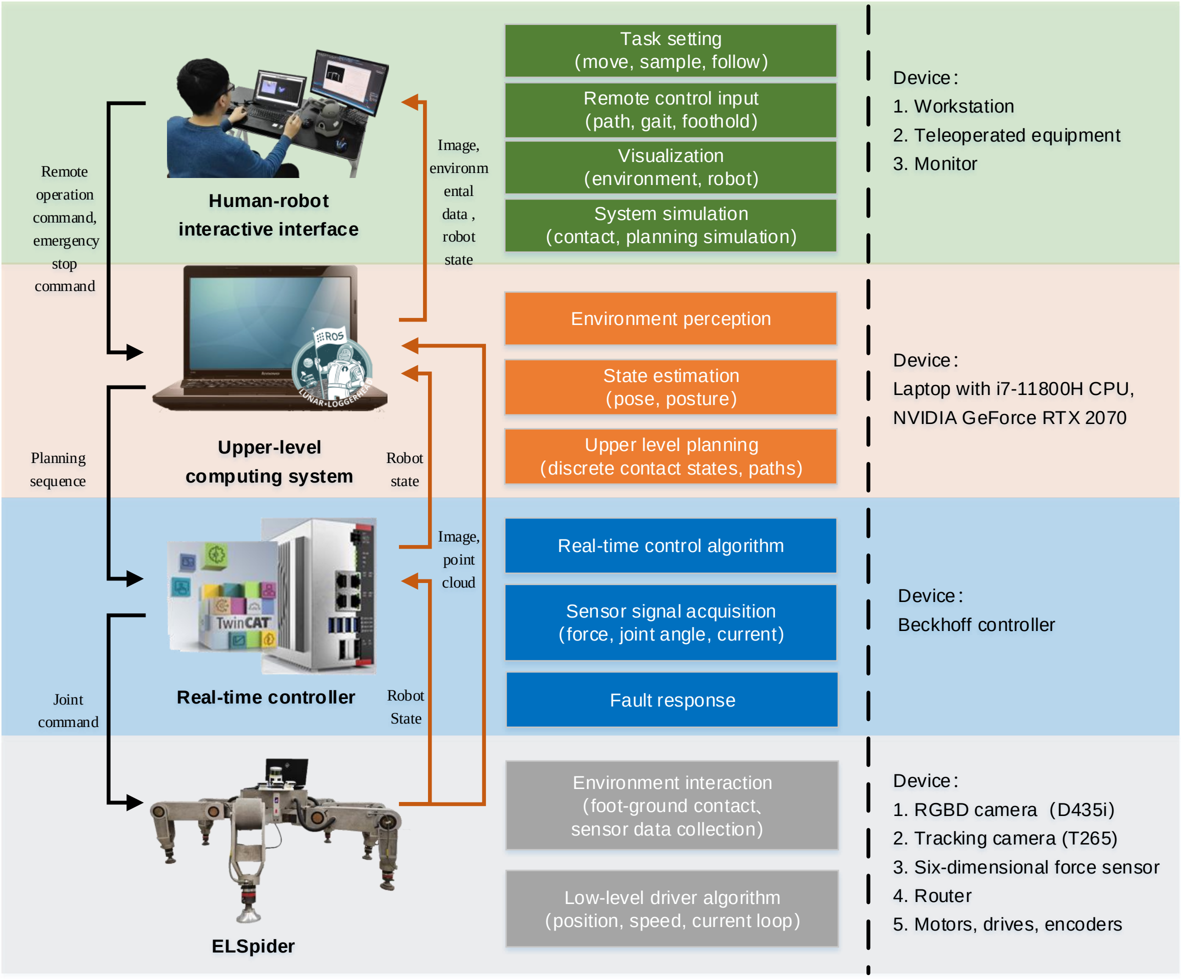

Figure S3. The hardware system.

S4. Algorithm Pseudocode

Algorithm S1 Train a Visual Feature Extraction Encoder

Collect N_{raw} images around the hexapod robot.
Create N_{patch} training samples by generating image patches using SLIC method.
while the loss is convergent **do**
    get a minibatch $\{x_k\}_{k=1}^{N}$ from training samples.
    for $k \in \{1, \ldots, N\}$ **do**
        get two new images $\tilde{x}_{2k-1}$ and $\tilde{x}_{2k}$ by augmentation method.
        Calculate their projection vectors $\mathbf{z}_{2k-1}$ and $\mathbf{z}_{2k}$.
    end for
    for $i, j \in \{1, \ldots, N\}$ **do**
        $s_{i,j} = \mathbf{z}_i^{\top} \mathbf{z}_j / \left(\|\mathbf{z}_i\| \left\|\mathbf{z}_j\right\| \right)$
    end for
    $\ell_{i,j} = -\log \frac{\exp\left(s\left(\mathbf{z}_i, \mathbf{z}_j\right)/\tau\right)}{\sum_{k=1}^{2N} \mathbf{1}_{[k \neq i]} \exp(\mathrm{s}(\mathbf{z}_i, \mathbf{z}_k)/\tau)}$
    Update parameters of the network through losses.
end while
return A visual feature extraction encoder

Algorithm S2 The Tactile Feature Extraction Method

Move a leg to the region of interest.
Control the foot to fall off until the foot force reaches a specified force threshold.
Control the foot moves tangentially on the ground keeping a fixed normal force.
Collect contact $\delta(t_i)$, $F_{\mathrm{T}}(t_i)$, $\delta(x_i)$, $F_{\mathrm{N}}(t_i)$.
for $i \in \{1, \ldots, N\}$ **do**
    $\min\limits_{k_{\mathrm{N}}, n_{\mathrm{N}}} \sum_{i=1}^{m} \left(k_{\mathrm{N}} \delta(t_i)^{n_{\mathrm{N}}} - F_{\mathrm{T}}(t_i)\right)^2$
    $\min\limits_{\mu, K} \sum_{i=1}^{m} \left(-\mathrm{th}\left(x(t_i) - x(0), 1.5K\right) \mu F_{\mathrm{N}} - F_{\mathrm{T}}(t_i)\right)^2$
end for
Calculate physical parameters k_{fri} and k_{soft}
$k_{\text{fri}} = \omega_1 \cdot \operatorname{atan}\left(\left|\frac{-\mu \cdot \mathrm{th}(3K, 1.5K) \mathrm{F_N}}{3K}\right|\right) + \omega_2 \cdot \mu \cdot \mathrm{F_N}$
$k_{\text{soft}} = \left(\frac{F_N'}{k}\right)^{1/n}$
return physical parameters k_{fri} and k_{soft}.

Algorithm S3 Create and Update the Associative Layer

Train the visual cognitive network using E-SOINN method in advance.
while True **do**
  Collect tactile parameters of terrains and update the tactile cognitive network. Then find the tactile type V_i from the network.
  Extract visual features of images collected by the hexapod robot and then find its visual cluster T_j from the visual cognitive network.
  if the visual cluster V_i does not appear in the associative layer **then**
    Create a new associative pair p_k between V_i and T_j in associative layer.
    $n_{\mathrm{a,k}} = n_{\mathrm{a,k}} + 1$
    $I_{\mathrm{spike}} = 1$
    $\gamma_{\mathrm{new},k} = \gamma_{\mathrm{last},k} + K_{\mathrm{LIF}}(-\gamma_{\mathrm{last},k} + I_{\mathrm{spike}})$
  end if
  if V_i appears in the associative layer and its corresponding tactile cluster in the layer is also T_j **then**
    Find the pair p_k contains V_i
    $n_{\mathrm{a,k}} = n_{\mathrm{a,k}} + 1$
    $I_{\mathrm{spike}} = 1$
    $\gamma_{\mathrm{new},k} = \gamma_{\mathrm{last},k} + K_{\mathrm{LIF}}(-\gamma_{\mathrm{last},k} + I_{\mathrm{spike}})$
  end if
  if V_i appears in the associative layer but its corresponding tactile cluster in the layer is not T_j **then**
    for each pair p_m contains V_i but without T_j **do**
      $n_{\mathrm{c,m}} = n_{\mathrm{c,m}} + 1$
      $I_{\mathrm{spike}} = -1$
      $\gamma_{\mathrm{new},m} = \gamma_{\mathrm{last},m} + K_{\mathrm{LIF}}(-\gamma_{\mathrm{last},m} + I_{\mathrm{spike}})$
    end for
    find or create associative layer p_k contains V_i and T_j
    $n_{\mathrm{a,k}} = n_{\mathrm{a,k}} + 1$
    $I_{\mathrm{spike}} = 1$
    $\gamma_{\mathrm{new},k} = \gamma_{\mathrm{last},k} + K_{\mathrm{LIF}}(-\gamma_{\mathrm{last},k} + I_{\mathrm{spike}})$
  end if
  for each pair p_l in he associative layer **do**
    if the activation intensity $\gamma_{\mathrm{new},l} < \gamma_{\mathrm{forget}}$ **then**
      Delete the pair.
    end if
    if the activation intensity $\gamma_{\mathrm{new},l} > \gamma_{\mathrm{activate}}$ **then**
      The pair is activated.
    end if
  end for
end while

Algorithm S4 Physical characteristics mapping method

if a new color frame is ready **then**
  Get a color image frame x from the camera installed on the hexapod robot.
  Generate image patches $x_1, x_2 \ldots x_M$ using SLIC method.
  $V_i = f_{\mathrm{VCN}}(x_m)$ //Search the classes of image patches from the visual cognitive network
  $T_j = f_{\mathrm{AL}}(V_i)$ //Get the tactile cluster of the terrain corresponding to each patch from the associative layer (AL).
  Then generate physical maps through $T_j(j = 1 \ldots N_{\mathrm{tactileCluster}})$ corresponding to $x_1, x_2 \ldots x_M$.
  Get the depth image of the same frame of the color image and generate semantic clouds through physical maps.
  Based on the pose of the depth camera in the world frame, project the semantic point cloud to the elevation maps.
end if
return physical characteristics maps

S5. Terrain Physical Parameters

Table S1. Physical Parameters of Terrains

	k_{N}	n_{N}	u	$K(mm)$	k_{soft}	k_{fri}
Marble Floor	76.64	0.92	0.32	1.30	2.84	1.30
Rubber Floor Mat	72.31	0.99	0.43	1.08	2.79	1.73
Blue Blanket	58.76	1.11	0.49	2.30	3.01	1.97
Sand	16.67	1.74	1.05	5.35	4.80	4.20
Ice	75.79	0.90	0.18	0.22	2.94	0.73
Soil	11.01	1.50	1.81	11.92	8.32	7.65
Snow	1.06	1.77	0.61	3.01	22.02	2.44
Asphalt	70.75	1.02	0.57	1.67	2.77	2.30
Sidewalk	72.85	0.98	0.55	1.49	2.80	2.21
Curbstone	71.64	0.94	0.31	0.87	2.98	1.26
Hard Floor	68.29	0.90	0.48	1.51	3.30	1.94
Grass	1.57	1.76	1.16	5.68	16.09	4.64
White Paint	70.64	0.93	0.54	0.90	3.06	2.18
Artificial Grass	7.38	1.73	0.73	4.56	6.73	2.93
Soft Foam Board	3.34	1.71	0.61	3.24	10.95	2.46
Blanket with Foam Board under it	3.56	1.70	0.47	2.26	10.69	1.90
Red(Blue) Mat glued to the ground	48.57	0.97	0.51	1.87	4.30	2.06
Red Mat	51.39	1.02	0.32	1.15	3.79	1.30
Blue Mat with Foam Board under it	4.86	1.58	0.48	0.98	10.51	1.94
Gravel	51.71	1.04	0.64	4.27	3.67	2.58
hard Soil	22.37	1.49	0.91	8.94	4.35	3.66

Table S2. Parameters for different feet with symmetrical stress distribution[13]

Model name	k_{N}	n_{N}	μ
Flat circular	$k_c\pi r + k_\varphi \pi r^2$	n	$\pi r^2 c/F_{\mathrm{TN}}^n + \tan(\varphi)$
Flat rectangular	$k_c a + k_\varphi ab$	n	$abc/F_{\mathrm{TN}}^n + \tan(\varphi)$
Cylindrical	$\sqrt{2r}k_c + \sqrt{2r}bk_\varphi$	$n+1/2$	$\sqrt{2r}bc/[\sqrt[2n+1]{(\sqrt{2r}k_c + sqrt2rbk_\varphi)F_{\mathrm{TN}}^n}] + \tan(\varphi)$
Spherical	$\pi k_c + \pi R k_\varphi$	$n+1$	$\pi Rc/[\sqrt[n+1]{(\pi k_c + \pi R k_\varphi)F_{\mathrm{TN}}^n}] + \tan(\varphi)$

Table S3. Associative Layer's Table of Outdoor Experiment 1

Tactile Class	Visual Class	n_a	n_c	γ
0	3(Sidewalk)	23	0	1
0	2(Asphalt)	15	0	0.999
0	7(Hard Floor)	10	0	0.999
1	5(Curbstone)	12	0	1
1	1(Curbstone)	4	0	0.936
1	4(Curbstone)	6	0	0.984
2	0(Glass)	15	0	1
2	6(Glass)	8	0	0.996

Table S4. Associative Layer's Table of Outdoor Experiment 2

Tactile Class	Visual Class	n_a	n_c	γ
0	1(Sidewalk)	3	0	0.875
0	3(Asphalt)	3	0	0.875
0	4(Asphalt)	3	0	0.875
0	7(Hard Floor)	3	0	0.875
1	9(Curbstone)	3	0	0.875
1	5(Curbstone)	3	0	0.875
2	6(White Paint)	3	0	0.875
2	2(Glass)	3	0	0.875
2	8(Glass)	3	0	0.875

Table S5. Associative Layer's Table of Indoor Experiment 1

Tactile Class	Visual Class	n_a	n_c	γ
1	1(Marble Floors)	3	0	0.875
2	5(Blue Blankets)	3	3	0.014
2	3(Rubber Floor Mats)	3	0	0.875
3	2(Artificial Grass)	3	0	0.875
4	4(Soft Foam Boards)	3	0	0.875
4	5(Blankets with Foam Boards under it)	3	0.014	0.875

Table S6. Associative Layer's Table of Indoor Experiment 2

Tactile Class	Visual Class	n_a	n_c	γ
1	1(Marble Floors)	3	0	0.875
2	2(Red Mats glued to the ground)	3	3	0.014
2	3(Blue Mats glued to the ground)	3	3	0.014
3	2(Red Mats)	3	0	0.875
4	3(Blue Mats with Foam Boards under it)	3	0	0.875

Table S7. The visual feature clustering accuracy table

Clustering Number	8	9	10	11	12
Clustering Accuracy	94.32%	95.13%	95.59%	96.03%	96.56%

Table S8. The tactile feature clustering accuracy table

Clustering Number	6	7	8	9	10
Clustering Accuracy	85.32%	83.32%	94.59%	97.12%	97.92%

Table S9. The physical parameter prediction accuracy table

	Gravel at Point B		Gravel at Point C		Gravel at Point D	
	K_soft	K_fri	K_soft	K_fri	K_soft	K_fri
The proposed method	92.36%	94.23%	90.79%	91.76%	89.58%	90.85%
The comparison method	92.36%	89.23%	-295.32%	40.15%	-275.67%	39.81%

Table S10. The comparison simulation results

	Completion Degree	Average Completion Time	The number of severe sink	The number of severe slip
The proposed method	100%	11.3min	0	0
The comparison method	65%	6.3min	13	74

S6. The impact of camera settings on prediction accuracy

Four different camera perspective settings are set: the overlooking angle of the camera (COA) is -60 degrees, and the flight height (FH) is 800mm; the COA is -30 degrees and FH is 800mm (the same as Hexapod's camera setting, as shown in Fig. S5); the COA is -30 degrees and FH is 2000mm; the COA is -60 degrees and FH is 2000mm. The surrounding images (the first indoor experimental scene) are collected by UAV with different camera settings, and images are segmented into patches as four training data set. For each set, a visual cognitive network (VCN) can be trained where each visual cluster corresponds to a terrain class. On the other hand, the images collected by the hexapod robot can be segmented into patches, and each patch is annotated by humans. Finally, the prediction (image patches predict their terrain classes) accuracy can be calculated,

$$\text{ClassPreAcc} = \frac{\sum_{i=1}^{N_{\text{patches}}} \mathbf{1}_{[L_{\text{predict}} = L_{\text{real}}]}}{N_{\text{patches}}} \tag{25}$$

Where L_{predict} is the prediction label; L_{real} is the real label; $\mathbf{1}_{[L_{\text{predict}} = L_{\text{real}}]}$ is an indicator function, its value is 1 when the prediction label is the same as the actual label, and otherwise, the value is 0; N_{patches} denotes the total number of test image patches.

The prediction accuracy results are shown in Table S11. We can see that when the camera settings are similar as that of hexapod's camera, the visual class prediction accuracy is around 90%; while when the camera settings are significantly different from that of hexapod's camera, the accuracy is extremely low. Therefore, the UAV are required to adopt hexapod camera settings in the proposed framework to keep a better prediction accuracy.

Table S11. The terrain class prediction results for different UAV camera settings

Camera Settings	COA(-60°), FH(800mm)	COA(-45°), FH(800mm)	COA(-30°), FH(800mm)	COA(-30°), FH(1000mm)	COA(-30°), FH(2000mm)	COA(-60°), FH(2000mm)
Prediction Accuracy	67.25%	86.25%	94.23%	90.58%	43.25%	32.68%

S7. Supplementary Figures

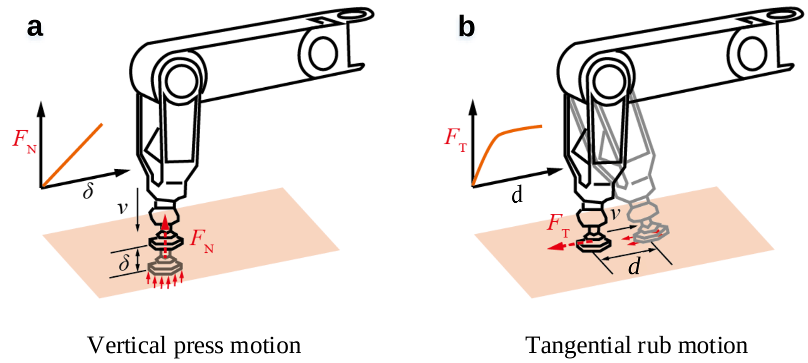

Figure S4. Exploratory tactile motions. a. The feet touch the ground slowly until the normal force reaches a threshold. **b**. The feet move tangentially while a fixed normal force is exerted by an admittance controller.

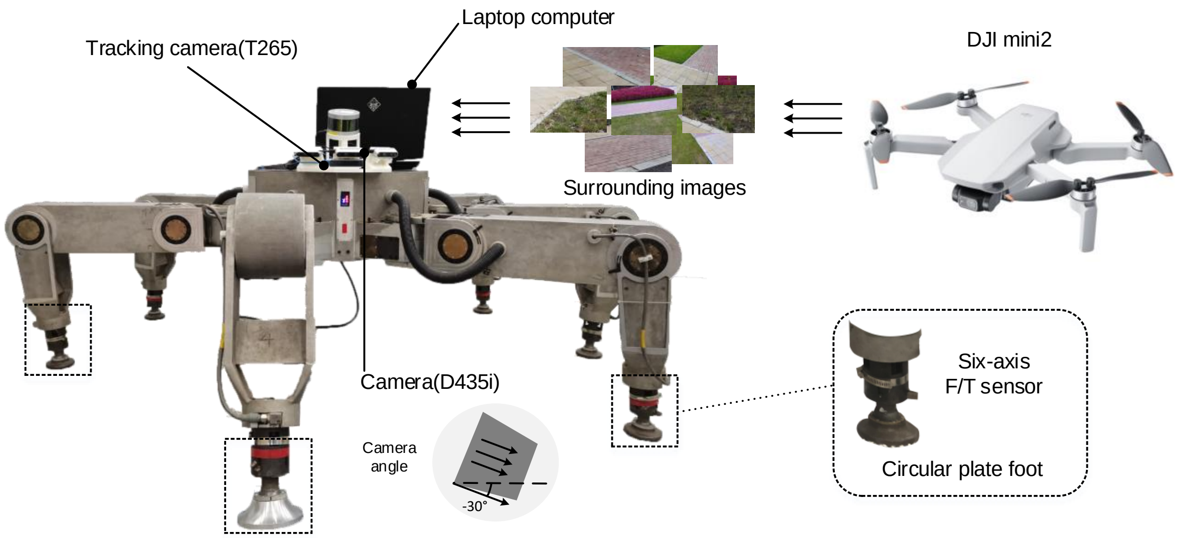

Figure S5. An overview of the main components of our robots.

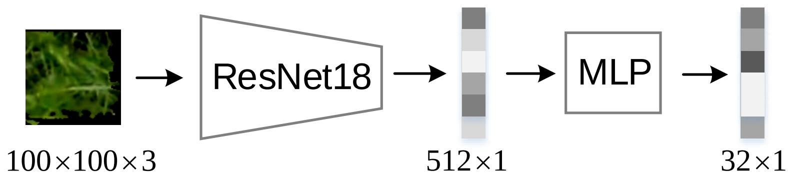

Figure S6. The input and output dimensions of the contrastive learning network.

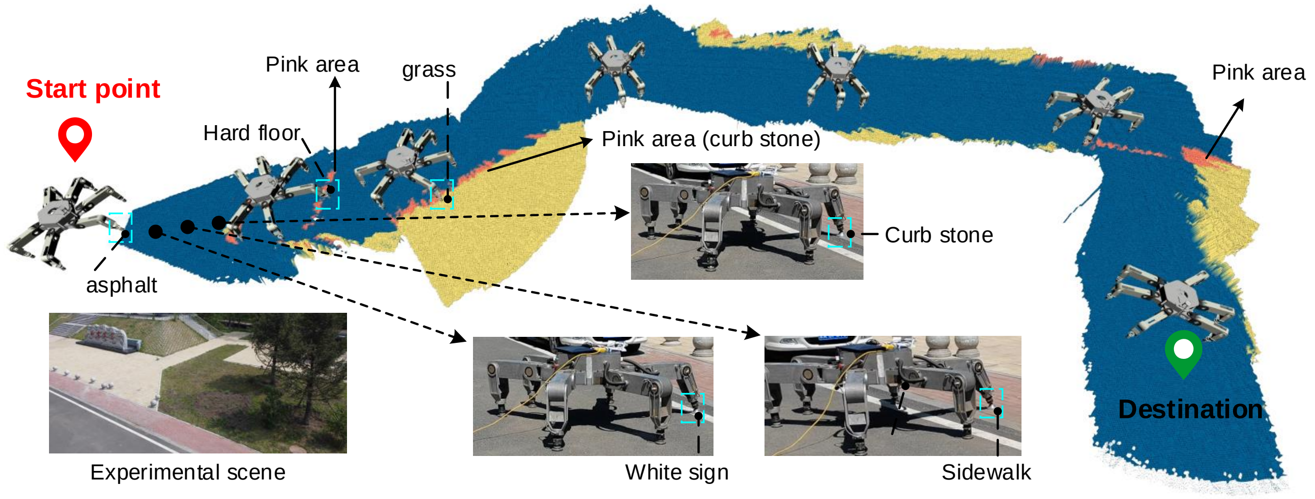

Figure S7. Navigation Experiments. The application of the prediction map on the robot navigation, where the predictive information is projected on the elevation map.

S8. Legends for Movies

Movie S1. The robot navigates outdoors to show the work process of our method.
https://youtu.be/FWd5xM3IZEg

Movie S2. The robot navigates indoors to show how the proposed method tackles cognitive conflicts.
https://youtu.be/316YsRkIOpU

Movie S3. Another indoor experiment to show the process of tackling cognitive conflicts and avoiding obstacles by selecting discrete footholds.
https://youtu.be/T8cwk3npeLE

Movie S4. Comparison experiments to show the advantage of the proposed method for robot navigation.
https://youtu.be/8Uv_Tnggy5g

Movie S5. Comparison simulations to show the advantage of the proposed method for robot navigation.
https://youtu.be/XrarC8MQ1U8

References

1. Han, Z. *et al.* White matter structural connectivity underlying semantic processing: evidence from brain damaged patients. *Brain* **136**, 2952–2965 (2013).
2. He, K., Fan, H., Wu, Y., Xie, S. & Girshick, R. Momentum contrast for unsupervised visual representation learning. In *Proceedings of the IEEE/CVF Conference on Computer Vision and Pattern Recognition*, 9729–9738 (2020).
3. Chen, T., Kornblith, S., Norouzi, M. & Hinton, G. A simple framework for contrastive learning of visual representations. In *International conference on machine learning*, 1597–1607 (PMLR, 2020).
4. Hjelm, R. D. *et al.* Learning deep representations by mutual information estimation and maximization. *arXiv preprint arXiv:1808.06670* (2018).
5. He, K., Zhang, X., Ren, S. & Sun, J. Deep residual learning for image recognition. In *Proceedings of the IEEE conference on computer vision and pattern recognition*, 770–778 (2016).
6. Oord, A. v. d., Li, Y. & Vinyals, O. Representation learning with contrastive predictive coding. *arXiv preprint arXiv:1807.03748* (2018).
7. Ding, L. *et al.* Pressing and rubbing: Physics-informed features facilitate haptic terrain classification for legged robots. *IEEE Robotics Autom. Lett.* **7**, 5990–5997 (2022).
8. Baird, A. A. *et al.* Frontal lobe activation during object permanence: Data from near-infrared spectroscopy. *NeuroImage* **16**, 1120–1126 (2002).
9. Furao, S., Ogura, T. & Hasegawa, O. An enhanced self-organizing incremental neural network for online unsupervised learning. *Neural Networks* **20**, 893–903 (2007).
10. Furao, S. & Hasegawa, O. An incremental network for on-line unsupervised classification and topology learning. *Neural networks* **19**, 90–106 (2006).
11. Ghosh-Dastidar, S. & Adeli, H. Spiking neural networks. *Int. journal neural systems* **19**, 295–308 (2009).
12. Izhikevich, E. M. Simple model of spiking neurons. *IEEE Transactions on neural networks* **14**, 1569–1572 (2003).
13. Ding, L. *et al.* Foot–terrain interaction mechanics for legged robots: Modeling and experimental validation. *The Int. J. Robotics Res.* **32**, 1585–1606 (2013).
